# Assessing the efficacy of target adaptive sampling long-read sequencing through hereditary cancer patient genomes

**DOI:** 10.1038/s41525-024-00394-z

**Published:** 2024-02-17

**Authors:** Wataru Nakamura, Makoto Hirata, Satoyo Oda, Kenichi Chiba, Ai Okada, Raúl Nicolás Mateos, Masahiro Sugawa, Naoko Iida, Mineko Ushiama, Noriko Tanabe, Hiromi Sakamoto, Shigeki Sekine, Akira Hirasawa, Yosuke Kawai, Katsushi Tokunaga, NCBN Controls WGS Consortium, Hatsue Ishibashi-Ueda, Tsutomu Tomita, Michio Noguchi, Ayako Takahashi, Yu-ichi Goto, Sumiko Yoshida, Kotaro Hattori, Ryo Matsumura, Aritoshi Iida, Yutaka Maruoka, Hiroyuki Gatanaga, Masaya Sugiyama, Satoshi Suzuki, Kengo Miyo, Yoichi Matsubara, Akihiro Umezawa, Kenichiro Hata, Tadashi Kaname, Kouichi Ozaki, Haruhiko Tokuda, Hiroshi Watanabe, Shumpei Niida, Eisei Noiri, Koji Kitajima, Yosuke Omae, Reiko Miyahara, Hideyuki Shimanuki, Shin-ichi Tsujimoto, Norio Shiba, Shuichi Ito, Teruhiko Yoshida, Yuichi Shiraishi

**Affiliations:** 1 Division of Genome Analysis Platform Development, National Cancer Center Research Institute, Tokyo, Japan; 2 Department of Pediatrics, Yokohama City University Hospital, Kanagawa, Japan; 3 Division of Genetic Medicine and Services, National Cancer Center Hospital, Tokyo, Japan; 4 Department of Molecular Pathology, National Cancer Center Research Institute, Tokyo, Japan; 5 Division of Laboratory Medicine, National Cancer Center Hospital, Tokyo, Japan; 6 Department of Clinical Genetics, National Cancer Center Research Institute, Tokyo, Japan; 7 Division of Molecular Pathology, National Cancer Center Research Institute, Tokyo, Japan; 8 Department of Clinical Genetics and Genomic Medicine, Okayama University Hospital, Okayama, Japan; 9 Genome Medical Science Project, Research Institute, National Center for Global Health and Medicine, Tokyo, Japan; 10 Central Biobank, National Center Biobank Network, Tokyo, Japan; 11 NCVC Biobank, National Cerebral and Cardiovascular Center, Osaka, Japan; 12 Medical Genome Center, National Center of Neurology and Psychiatry, Tokyo, Japan; 13 Department of Bioresources, Medical Genome Center, National Center of Neurology and Psychiatry, Tokyo, Japan; 14 Department of Clinical Genome Analysis, Medical Genome Center, National Center of Neurology and Psychiatry, Tokyo, Japan; 15 NCGM Biobank, National Center for Global Health and Medicine, Tokyo, Japan; 16 AIDS Clinical Center, National Center for Global Health and Medicine, Tokyo, Japan; 17 Department of Viral Pathogenesis and Controls, Research Institute, National Center for Global Health and Medicine, Chiba, Japan; 18 Center for Medical Informatics and Intelligence, National Center for Global Health and Medicine, Tokyo, Japan; 19 National Center for Child Health and Development, Tokyo, Japan; 20 Center for Regenerative Medicine, National Center for Child Health and Development, Tokyo, Japan; 21 Department of Maternal–Fetal Biology, National Center for Child Health and Development, Tokyo, Japan; 22 Department of Genome Medicine, National Center for Child Health and Development, Tokyo, Japan; 23 Research Institute, National Center for Geriatrics and Gerontology, Aichi, Japan

**Keywords:** Genetics research, Genomics, Data processing, Clinical genetics

## Abstract

Innovations in sequencing technology have led to the discovery of novel mutations that cause inherited diseases. However, many patients with suspected genetic diseases remain undiagnosed. Long-read sequencing technologies are expected to significantly improve the diagnostic rate by overcoming the limitations of short-read sequencing. In addition, Oxford Nanopore Technologies (ONT) offers adaptive sampling and computationally driven target enrichment technology. This enables more affordable intensive analysis of target gene regions compared to standard non-selective long-read sequencing. In this study, we developed an efficient computational workflow for target adaptive sampling long-read sequencing (TAS-LRS) and evaluated it through application to 33 genomes collected from suspected hereditary cancer patients. Our workflow can identify single nucleotide variants with nearly the same accuracy as the short-read platform and elucidate complex forms of structural variations. We also newly identified several SINE-R/VNTR/Alu (SVA) elements affecting the *APC* gene in two patients with familial adenomatous polyposis, as well as their sites of origin. In addition, we demonstrated that off-target reads from adaptive sampling, which is typically discarded, can be effectively used to accurately genotype common single-nucleotide polymorphisms (SNPs) across the entire genome, enabling the calculation of a polygenic risk score. Furthermore, we identified allele-specific *MLH1* promoter hypermethylation in a Lynch syndrome patient. In summary, our workflow with TAS-LRS can simultaneously capture monogenic risk variants including complex structural variations, polygenic background as well as epigenetic alterations, and will be an efficient platform for genetic disease research and diagnosis.

## Introduction

The advances in sequencing technology have improved the rates of diagnosis of genetic diseases. However, even with whole genome sequencing analysis, the diagnosis rate is still less than half^1^. There has been a lot of interest in long-read sequencing technologies in recent years due to their ability to solve some of the issues related to short-read technologies such as ambiguous alignments on repeat regions and improving SV detection performance^2–4^. In addition, long-read allows us to execute phasing over an extensive range and allows for a deeper understanding of the nature of genetic mutations in clinically relevant genes^5,6^. Being able to perform phasing also enables the division of reads by haplotype, leading to the execution of accurate mutation calling^7,8^. Furthermore, long-read sequencing technology also provides DNA modification information, such as 5-methylcytosine and 5-hydroxymethylcytosine, by the signal generated during the sequence of naïve DNA molecules without additional manipulation^8–10^. Long-read sequencing was, however, plagued by both high error rates and high costs. In order to alleviate these problems, a variety of targeted long-read sequencing has been developed. Examples of these include PCR enrichment^11,12^ and Cas9 target cleavage^13–15^. However, it requires a substantial amount of time during sample preparation.

Recent Oxford Nanopore Technologies (ONT) sequencing instruments are equipped with adaptive sampling functions, which accept or reject DNA molecules based on real-time alignment to sequences in a target region^16–18^. One major advantage of target adaptive sampling long read sequence (TAS-LRS) is that the user only needs to specify the coordinates of the target area, and no prior sample prep is required. The performance of adaptive sampling has been demonstrated in various applications such as Mendelian variant detection, elucidation of complex chromosomal rearrangements, and rapid neurooncology diagnostics^5,19–21^. Still, the development of a workflow that can systematically identify clinically significant variants is still in its infancy.

**Fig. 1:**
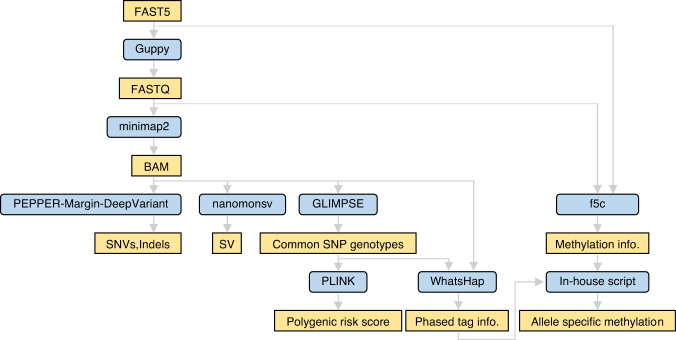
Overview of the workflow for adaptive sampling using nanopore sequencing. The FAST5 files were base-called using highaccuracy model in Guppy, followed by alignment of all FASTQ files using minimap2. De novo detection of SNVs/Indels were performed through PEPPER-Margin-DeepVariant, while SVs were identified using nanomonsv. Common SNP genotypes were called using GLIMPSE. For downstream analysis, the polygenic risk score was calculated with PLINK using genotyping results obtained from GLIMPSE. In allele-specific methylation analysis, each read in the BAM file was assigned to its respective haplotype using WhatsHap, based on the genotyping results obtained from GLIMPSE. The methylation calling for each haplotype was performed using f5c, and the identification of aberrantly methylated genes was performed using an in-house script.

We have developed a workflow designed for TAS-LRS sequencing analysis (Fig. 1). Our pipeline can precisely detect SNVs and structural variations (SVs) including mobile element insertions such as LINE1, Alu, and SINE-R/VNTR/Alu (SVA). In addition, we have also automated the subsequent detection of pathogenic mutations, making it possible to list candidate variants immediately. In addition, our workflow offers an allele-specific methylation analysis.

Moreover, we demonstrated that fairly accurate genome-wide common SNP genotyping is possible by making the most of off-target data from TAS-LRS. Recently, various analyses have shown that not only monogenic pathogenic variants but also polygenic architecture, which is exemplified by polygenic risk score (PRS), have a significant impact on many diseases^22–25^. The calculation of PRS has been typically performed from SNP array or high-coverage whole genome sequencing data. In addition, methods such as low-pass whole genome sequencing with an average sequencing depth of around 1.0× and genotyping from off-target reads in panel sequencing have recently been proposed as a cost-effective option^26–28^. Since adaptive sampling TAS-LRS produces low-coverage sequence data at off-target regions throughout the genome, it would be a reasonable attempt to calculate PRS using them.

We applied our workflow to the TAS-LRS sequencing data from clinically suspected hereditary cancer syndrome patients with the goal of identifying causative variants not found by existing clinical tests, such as panel sequencing, and elucidating the detailed structure of partially detected SVs. Furthermore, we performed the evaluations on SNVs/Indels calling and common SNP genotyping through the comparison with the whole genome sequencing data from short-read sequencing (WG-SRS) data as a benchmark.

## Results

### Selection of 33 patients with suspected hereditary cancer syndromes

In this study, we focused on 33 patients with clinically suspected hereditary cancer syndromes, consisting of 11 familial adenomatous polyposis (FAP), 4 hereditary breast and ovarian cancer (HBOC), 4 retinoblastoma (RB), 4 Li–Fraumeni syndrome (LFS), 2 Lynch syndrome (LS), and 8 other syndromes (Table 1). Two FAP patients had signals of SVs in the *APC* gene from prior analyses (short-read target sequencing (NCC Oncopanel Test^29,30^), or multiplex ligation-dependent probe amplification (MLPA)), and were analyzed by TAS-LRS to elucidate the detailed form of the SVs precisely. In the two patients with RB, the presence of SVs involving the *RB1* gene was known in advance by FISH and other methods, and TAS-LRS analysis was performed to identify the location of the breakpoints accurately. For 22 patients, high-coverage (range: 26.3×–31.7×) whole genome sequencing data by Novaseq 6000 platform were available.

**Table 1:** The cohort of patients who were sequenced with target adaptive sampling long-read sequencing in this study

Sample ID	Clinical diagnosis	WGS	WTS	NCC oncopanel test	G-banding or FISH	MLPA or Sanger	Previous result of genetic analysis
S1	LS	◯	–	◯	–	–	No pathogenic variants detected.
S2	FAP	◯	◯	◯	–	◯	A reciprocal translocation with a breakpoint in *APC* gene was detected by WGS.
S3	RB	◯	◯	◯	◯	◯	A Reciprocal translocation with a breakpoint in *RB1* gene was detected by FISH and WGS.
S4	FPC	◯	–	◯	–	◯	No pathogenic variants detected.
S5	FAP	◯	◯	◯	–	◯	No pathogenic variants detected.
S6	FAP	◯	◯	◯	–	◯	No pathogenic variants detected.
S8	FAP	◯	◯	◯	–	◯	No pathogenic variants detected.
S9	MEN1	◯	◯	◯	–	◯	No pathogenic variants detected.
S11	MEN2	◯	◯	◯	–	◯	No pathogenic variants detected.
S12	LFS	◯	◯	◯	–	◯	No pathogenic variants detected.
S14	LFS	◯	–	◯	–	◯	No pathogenic variants detected.
S15	HBOC	◯	–	◯	–	–	No pathogenic variants detected.
S16	FAP	◯	◯	◯	–	◯	No pathogenic variants detected.
S19	HAML	◯	◯	◯	–	◯	No pathogenic variants detected.
S20	FAP	◯	◯	◯	–	◯	No pathogenic variants detected.
S21	PHTS	◯	–	◯	–	◯	No pathogenic variants detected.
S22	HBOC	◯	◯	◯	–	◯	No pathogenic variants detected.
S23	RB	◯	–	◯	◯	◯	No pathogenic variants detected.
S24	RB	◯	◯	◯	◯	◯	No pathogenic variants detected.
S25	FPC	◯	◯	◯	–	–	No pathogenic variants detected.
S26	FAP	◯	◯	◯	–	◯	No pathogenic variants detected.
S27	LFS	◯	◯	◯	–	◯	No pathogenic variants detected.
S28	PJS	–	–	◯	–	◯	No pathogenic variants detected.
S29	ICC	–	–	◯	–	–	No pathogenic variants detected.
S30	HBOC	–	–	◯	–	◯	No pathogenic variants detected.
S31	LFS	–	–	◯	–	◯	No pathogenic variants detected.
S32	FAP	–	–	◯	–	◯	No pathogenic variants detected.
S33	LS	–	–	◯	–	◯	No pathogenic variants detected.
S34	HBOC	–	–	◯	–	◯	No pathogenic variants detected.
S35	HDGC	–	–	◯	–	◯	No pathogenic variants detected.
S36	FAP	–	◯	◯	–	◯	No pathogenic variants detected.
S37	FAP	–	–	◯	–	◯	A complex structural variation involving *APC* gene was suspected by MLPA.
S38	RB	–	–	◯	◯	◯	A deletion of the *RB1* gene was suspected by MLPA.

This table details the cohort of patients who were sequenced with target adaptive sampling long-read sequencing in this study. Columns labeled WGS, WTS, FISH, and MLPA indicate whether whole genome sequencing, whole transcriptome sequencing, fluorescence in situ hybridization, and multiplex ligation-dependent probe amplification were performed or not, respectively. The “Clinical diagnosis” column employs abbreviations as follows: *FAP* familial adenomatous polyposis, *FPC* familial pancreatic cancer, *HAML* hepatic angiomyolipoma, *HBOC* hereditary breast and ovarian cancer, *HDGC* hereditary diffuse gastric cancer, *ICC* intra-hepatic cholangiocarcinoma, *LFS* Li–Fraumeni syndrome, *LS* lynch syndrome, MEN1 multiple endocrine neoplasia type 1, MEN2 multiple endocrine neoplasia type 2, PHTS PTEN Hamartoma tumor syndrome, PJS Peutz–Jeghers syndrome, and *RB* retinoblastoma.

### Summary of sequencing statistics

We curated 147 cancer predisposition genes from the literature. To generate the BED file specifying a target region, the maximal regions of all the corresponding transcripts registered in the GENCODE Basic gene annotation Release 38 with a margin of 10 kbp for each gene were set. For the *APC* gene, two starting exons, Exon 1A and Exon 1B, have been identified, and both are implicated to have important functions^31,32^. However, transcripts including Exon 1B were not included in the GENCODE Basic gene annotation release 38. Therefore, we manually expanded the *APC* region to include Exon 1B. The resulting target region size was 16,122,639 bp (see Supplementary Data 1). For these target regions, we performed sequencing with GridION (see the “Methods” section for details).

**Fig. 2:**
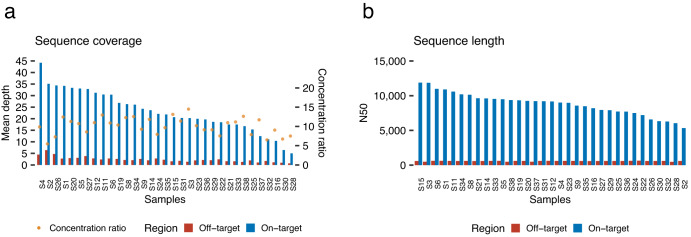
Quality check summary of TAS-LRS sequencing data. **a** Sequence coverage of on-target and off-target regions and the concentration ratio (ratio of on-target to off-target coverage) for each sample. Samples were ordered by on-target coverage. **b** N50 statistics calculated for on-target and off-target regions for each sample. Samples were ordered by on-target N50 values.

The median depth of on-target and off-target regions were 21.9 (range: 5.0–44.2) and 2.1 (range: 0.67–6.3), respectively. The median enrichment of the on-target regions compared to the off-target regions was 10.4 (range: 5.5–14.5) (Fig. 2a). 17 out of 33 samples had 20× or greater depth of coverage over 50% of the target regions (Supplementary Fig. 1). After reviewing the coverage for each gene, we found no gene with exceptionally low coverage (Supplementary Fig. 2). Two samples had an average depth of coverage of <10×, compromising the accuracy of subsequent analyses, including mutation detection. The read N50 of on-target and off-target regions were 9159 (range: 5330–11,885) and 598 (range: 475–635) (Fig. 2b), respectively.

### Evaluation of SNVs/Indels detection

**Fig. 3:**
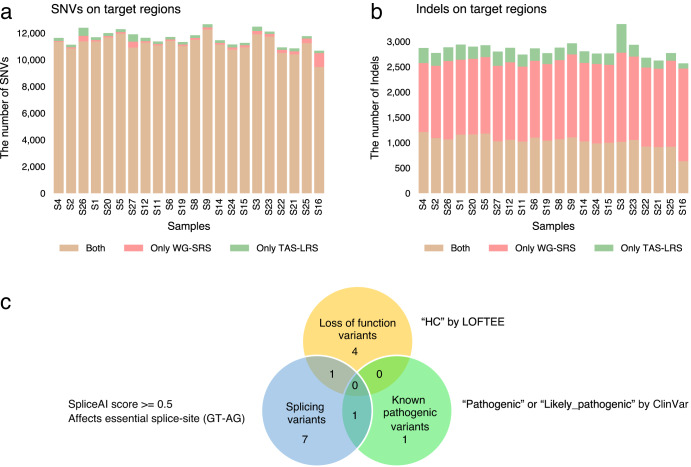
Summary of SNVs/Indels detected in the target region. **a**, **b** The number of SNVs (**a**) and Indels (**b**) for each sample stratified by whether they were detected by TAS-LRS, WG-SRS, or both. **c** Venn diagram showing the categories of putative pathogenic variants identified (known pathogenic, loss-of-function, and splicing variants). See also Supplementary Fig. 3.

A median of 15,398 (range: 8,633–16,982) SNVs/Indels were detected in the target regions in TAS-LRS. To evaluate the accuracy of the detection of SNVs/Indels, we compared the detection between TAS-LRS and high-coverage whole-genome short-read sequence (WG-SRS) for 22 cases where matched WG-SRS data were available. For SNVs, the results of the TAS-LRS and WG-SRS were fairly consistent. Setting the SNVs identified from the WG-SRS platform as golden datasets, the median recall and precision for TAS-LRS were 98.8% (range: 90.0–99.4%) and 98.2% (range: 94.9–98.5%), respectively (Fig. 3a). The recall of SNVs decreased as the depth of coverage decreased (Supplementary Fig. 3), as demonstrated by one case with very low sequence coverage. On the other hand, regarding Indels, the results for the TAS-LRS and WG-SRS were largely different, probably because slippage errors are much more abundant in Oxford Nanopore Technologies compared to the Illumina-based short-read platform (Fig. 3b).

### Extraction of candidate pathogenic SNVs/Indels

From the list of SNVs/Indels detected in the target regions, we extracted candidate pathogenic variants by combining various information such as population allele frequencies, registries of known relationships among variants and diseases (ClinVar^33^), and functional prediction tools (see the “Methods” section for detail), and 14 putative pathogenic variants were identified (Fig. 3c, Supplementary Fig. 4). Two variants were known pathogenic variants registered in ClinVar. Five were high confidence loss of function variants according to LOFTEE^34^. Nine were predicted to cause aberrant splicing. There were two variants belonging to two categories (Supplementary Data 2). Although these putative pathogenic variants were generally detected by previously performed analyses (NCC Oncopanel Test and WG-SRS), we would like to highlight some potential pathogenic SNVs below.

In a clinically suspected multiple endocrine neoplasia type 2 (MEN2) case (S11), we identified a potential splicing variant in the *EPCAM* gene (c.556-14A>G), which has been registered as “pathogenic” with two stars in the ClinVar (Supplementary Fig. 5a). Furthermore, we identified polymorphisms G691S/S904S in the *RET* gene, whose modifier effects on MEN2 have been investigated in several previous studies^35,36^. Therefore, a combination of heterogeneous effects of various mutations might produce symptoms in this patient.

In a patient with clinically suspected Hereditary Diffuse Gastric Cancer (S35), we detected a G258E missense variant in the *MUTYH* gene, which is annotated as “Pathogenic/Likely pathogenic” with two stars in ClinVar (Supplementary Fig. 5b). Although impaired glycosylase activity was demonstrated by functional assay for this variant^37^, *MUTYH* is generally considered to be autosomal recessive, and the other mutation has not yet been detected. The association between this mutation and the disease needs to be further investigated.

We also identified a missense mutation in A189V in the *TP53* gene in a clinically suspected LFS patient (S27), which is registered in ClinVar as “Conflicting interpretations of pathogenicity .” This variant showed relatively high minor allele frequency in East Asian cohorts (1.77 × 10^−3^ in ToMMo 38KJPN database^38^, and 5.46 × 10^−4^ in Korea 1K) compared to worldwide cohorts (6.57 × 10^−6^ in gnomAD v3.1.2^34^). The odds ratios for this variant were modest (1.7–1.8) in the previous Japanese breast and colorectal cancer cohort study^39,40^. Therefore, the A189V variant in *TP53*, if any, would have only low penetrance pathogenicity.

### Overview of potentially functional SV detection

Application of nanomonsv and a false-positive SV elimination filter (see the “Methods” section for details) yielded a total of 44 SVs. Furthermore, subsequent putative pathogenic SV extraction (in short, extracting SVs disrupting coding sequences, see the “Methods” section for detail) nominated 12 SV breakpoint junctions, two of which were identified as single breakend SV and were in the intron region of the *APC* gene (Supplementary Data 3 and 4). In fact, all of them involved *RB1* in two RB patients (S3 and S38) or *APC* in two FAP patients (S2 and S37) (Supplementary Figs. 6 and 7). In the following, we describe a novel clarification provided by TAS-LRS compared to previous tests.

**Fig. 4:**
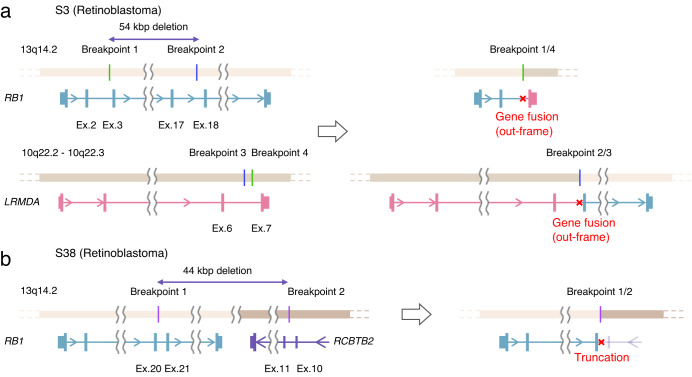
Schematic representation of structural variations of the *RB1* gene in two patients with Retinoblastoma. **a** A balanced translocation involving *RB1* detected in S3 consists of two interchromosomal junctions. One junction connects breakpoint 1 (in the 2nd intron of the *RB1* gene) and breakpoint 4 (in the 6th intron of the *LRMDA* gene), and the other junction juxtaposes breakpoint 3 (in the 17th intron of the *RB1* gene) and breakpoint 4 (in the 6th intron of the *LRMDA* gene). Approximately 54 kbp region between breakpoint 1 and breakpoint 2 in the *RB1* gene was deleted. **b** A deletion spanning a 44 kbp region spanning from the 20th intron of the *RB1* gene to the 10th intron of the *RCBTB2* gene.

Our analysis resolved a complex intrachromosomal balanced translocation affecting the *RB1* gene and the *LRMDA* gene and identified precise breakpoints, consistent with the one identified by WG-SRS analysis (Fig. 4a). S38 had been inferred to have deletions of exons 21–27 (last exon) via MLPA. We showed a large deletion of 44,362 bp extending from the 20th intron of the *RB1* gene to the adjacent *RCBTB2* gene with exact breakpoint coordinates (Fig. 4b).

Multi-gene panel testing and MLPA had shown partially identified signals indicating SVs on *APC* in S37. However, the overall structure had not yet been elucidated. Our analysis based on TAS-LRS detected two SV breakpoint junctions constituting reciprocal inversions accompanied by 130 kbp deletion. In the other patient with FAP (S2), a reciprocal translocation involving a breakpoint in the *APC* gene was identified (Supplementary Fig. 8).

### Two SVA insertions affecting *APC* in FAP patients

**Fig. 5:**
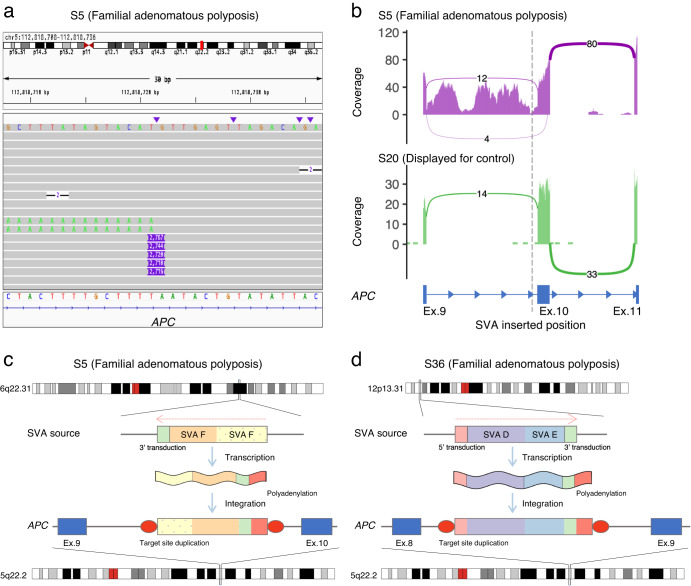
Details of SVA-derived insertion into the intronic region of the *APC* gene in two patients with familial adenomatous polyposis. **a** The IGV displayed long-read sequencing data and transcript sequencing data showing an SVA-derived insertion of 2731 bp in the 9th intron of the *APC* gene. **b** The whole transcriptome sequence showed specific intron retention at the near exon–intron boundary. **c** An SVA inserted into the 9th intron of the *APC* gene in patient S5, derived from two concatenated human-specific subfamily SVA_F elements located at 6a22.31, which undergoes 5´ truncation and poly(A) tail addition prior to insertion. **d** An SVA inserted into the 8th intron of the *APC* gene in patient S36, derived from concatenated SVA_D and SVA_E elements located at 12p13.31, which undergoes poly(A) tail addition prior to insertion.

We further searched the list of 44 SVs in the phase immediately following the false-positive SV elimination filter, focusing in particular on those affecting genes that are well known to be associated with the predicted diseases. This search found a 2731 bp insertion, which matched to SINE-R/VNTR/Alu (SVA) by RepeatMasker^41^, in the 9th intron of the *APC* genes in a patient of strongly suspected FAP (Fig. 5a). SVA is a class of recent mobile elements found only in primates. Mobile element insertions including SVA can cause disease typically by inactivating gene function through abnormal splicing^42,43^. Previous studies have found a number of diseases derived from SVA insertions. Although LINE1, another class of mobile element insertion, has been identified in familial adenomatous polyposis^44–46^, there has been no study that finds SVA insertion in FAP patients as far as we know. The whole transcriptome sequence performed on the same patient revealed significant and specific intron retention at the near exon–intron boundary, implicating the pathogenicity of the SVA insertion in this patient (Fig. 5b).

Next, we investigated the source site by alignment of the polished SVA sequence (inferred by Racon^47^ integrated into nanomonsv) to the reference genome using BLAT^48^. The SVA strongly matched the sequence of a region on chromosome 6 (chr6:122,847,699–122,850,317), consisting of two adjacent SVA_F sequences (chr6:122,849,195–122,850,317, chr6:122,847,781–122,849,162) and 82 bp 3′ transduction (chr6:122,847,699–122,847,780), accompanied by a 24 bp polyadenylation tail and 14 bp target site duplication (Fig. 5c). The 3′ transduction also contained the predicted conserved polyadenylation signal AATAAA. Since the 5′ end of SVA_F ((CCCTCT)_*n*_ repeats that are necessary for retrotransposition^49,50^) is truncated, the inserted SVA sequence into *APC* is thought to have already lost its trans-mobility capability.

Motivated by this finding, we further investigated other mobile element insertions by manually investigating the BAM files and identified another 2678 bp SVA insertion in the 8th intron of the *APC* gene in another FAP patient (Supplementary Fig. 9). This inserted sequence was inferred to be derived from a region of chromosome 12 (chr12:8,624,237–8,626,878), which consists of SVA_D (chr12:8,624,321–8,625,515), SVA_E (chr12: 8,625,529–8,626,786), and 91 bp of the 3′ transduction (which included polyadenylation signal), followed by 55 bp polyadenylation tail and 15 bp target site duplication (Fig. 5d).

### Verification of genome-wide common SNP genotyping from sequence data from off-target regions

The TAS-LRS provides low-coverage sequencing data even in the off-target regions. There have been several attempts to genotype SNPs genome-wide using off-target sequence data. However, these studies were mostly performed using short-read platforms with few sequence errors, and only a few attempts have been made on error-prone long reads^51^. Here, we focused on genotyping of common SNPs across the genome (mostly off-target regions), using data from long-read while making the most of off-target reads that would otherwise be discarded.

**Fig. 6:**
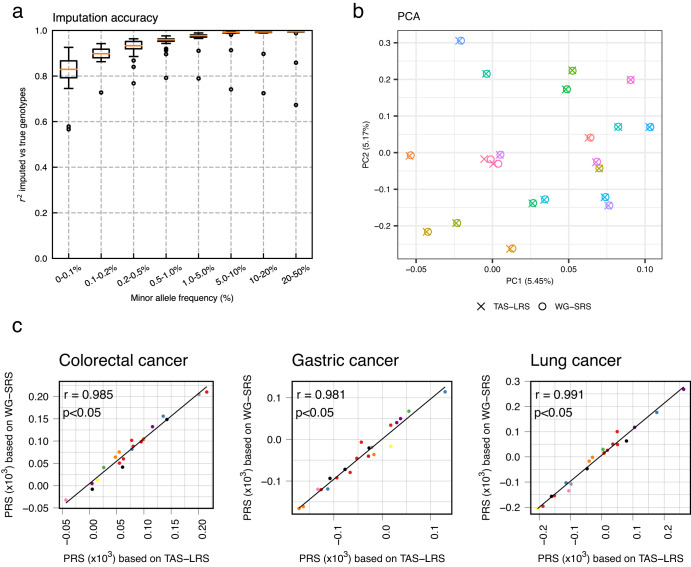
Comparison of genome-wide common SNP genotyping by TAS-LRS (imputation of low-coverage off-target sequencing data using GLIMPSE) compared to WG-SRS (direct variant calling on high-coverage whole-genome sequencing data by GATK). **a** Imputation accuracy of TAS-LRS was measured on chromosome 1 for each minor allele frequency range. Genotyping by WG-SRS was used as the golden standard. See also Supplementary Fig. 9. Box plots show medians (lines), interquartile ranges (IQRs; boxes), ±1.5 × IQRs (whiskers), and outliers (dots). **b** PCA of genotype results from both TAS-LRS and WG-SRS for each individual (distinguished by color). Pairs of the same individuals are clearly clustered, indicating that the batch effect of the difference between the TAS-LRS and WG-SRS platforms has effectively disappeared. One outlier sample that could have originated from different ancestries was excluded. See also Supplementary Fig. 10. **c** Comparison of PRSs for three cancers calculated from the genotype by TAS-LRS (*X*-axis) and WG-SRS (*Y*-axis). Each point indicates each sample and each color indicates each syndrome name (red: Familial adenomatous polyposis, blue: Familial pancreatic cancer, green: Hepatic angiomyolipoma, purple: Hereditary breast and ovarian cancer, orange: Li–Fraumeni syndrome, yellow: Lynch syndrome, brown: multiple endocrine neoplasia type 1, pink: multiple endocrine neoplasia type 2, gray: PTEN hamartoma tumor syndrome, black: Retinoblastoma).

We performed genome-wide common SNP genotyping on TAS-LRS using GLIMPSE^28^ with a reference panel consisting of 8570 Japanese genomes from the National Center Biobank Network (NCBN)^52^ project as well as 3202 genomes from 1000 genomes. The total number of SNPs in the panel was 39,201,938. The number of SNPs genotyped by GLIMPSE for each patient was a median of 1,750,570 (range:1,509,006–1,757,025). The median concordance with WG-SRS was 99.8% (range: 92.0–99.9%) (Fig. 6a, Supplementary Fig. 10), showing that common SNP genotyping utilizing low-coverage sequencing reads in the off-target region is fairly accurate in most cases.

We evaluated the performance of downstream analyses using genotyping results obtained from the above procedure. The result of the principal component analysis (PCA) for the genotype data from both TAS-LRS and WG-SRS showed that identical individuals were strongly clustered, indicating that the batch effect from the platform difference was effectively removed (Fig. 6b, Supplementary Fig. 11). Next, we calculated a polygenic risk score (PRS) using GWAS summary statistics data^53^ related to various cancer types in the Japanese population and examined the correlation between the scores from TAS-LRS and WG-SRS. PRS calculated by the two platforms showed a strong correlation. The median Pearson correlation for the 12 carcinomas was 0.989 (range: 0.976–0.997) (Fig. 6c, Supplementary Fig. 12). These results indicate that genome-wide common SNP genotyping using discarded off-target reads from TAS-LRS is sufficiently accurate for many downstream analyses, such as PRS calculation.

To further validate our findings, we assessed genotyping accuracy using low-coverage ONT sequencing data with the widely recognized HG001 sample, employing down-sampling. We consistently demonstrated that precise genotyping is possible from low-coverage ONT sequencing data (Supplementary Fig. 13). At the same time, we observed that genotyping accuracy changes with depth, showing significant differences between 1× and 2×. This observation suggests potential advantages in incorporating periods without specific target sampling in adaptive sampling. Furthermore, we also validated using another tool called QUILT^51^. The results were of comparable accuracy to GLIMPSE. However, there is still room for optimization in the future, such as the choice of software, parameter tuning, and refining the reference panel.

### *MLH1* epimutation in a patient of Lynch syndrome identified via allele-specific methylation analysis

An important advantage of the Oxford Nanopore Technologies sequencing data is that epigenetics modifications such as methylation can be obtained for each sequence read and position. Furthermore, by combining the genome-wide genotype obtained in the previous section, which also includes phasing information, it is possible to classify each sequence read by haplotype and obtain allele-specific methylation information. We have implemented a workflow to automatically identify allele-specific methylation regions where the methylation ratios significantly differ from those of 10 control samples (see the “Methods” section for details).

**Fig. 7:**
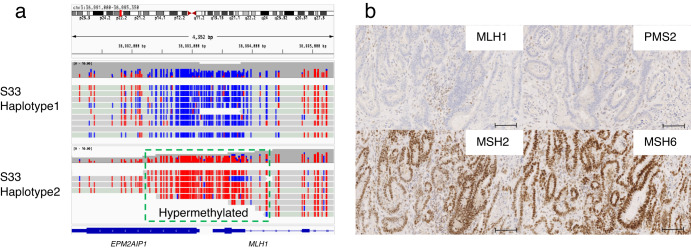
A case of an *MLH1* epimutation in a patient with LS. **a** Alignment view of around the promoter region of the *MLH1* gene. Each read was classified as haplotype 1 or 2 using Whatshap software. The CpG sites of each read are colored red if methylated and blue if not. It can be clearly seen that methylation is increased specifically for haplotype 2. **b** Immunohistochemical staining for DNA mismatch repair protein performed on cancer tissue from patient S33. Loss of immunohistochemical expression of MLH1/PMS2 was observed. Scale Bar = 100 μm.

Our analyses revealed constitutional *MLH1* epimutation^54,55^ in a patient diagnosed with Lynch syndrome. Although this patient tested positive for microsatellite instability using tumor samples, no germline mutation was detected in the multi-gene panel testing. As shown in Fig. 7a, we visualized the allele-specific methylation status of this patient using our workflow. Our workflow revealed that one allele of the *MLH1* exhibited hypermethylation in the promoter region, suggesting that the *MLH1* gene expression is reduced in one allele. Based on the above, the patient was suspected o*f MLH1* epimutation for the first time. Through immunohistochemistry, we observed not only the loss of MLH1 but also of PMS2 protein, recognizing the typical pattern of Lynch syndrome due to MLH1 loss (Fig. 7b). This is because PMS2 forms a heterodimer with MLH1 and is more prone to degradation when MLH1 is absent.

Also, we detected a patient with FAP, which showed abnormal hypermethylation in the first intron region of the *BARD1* gene. However, their relevance to the disease remains to be investigated (Supplementary Fig. 14).

## Discussion

In this study, we demonstrated that TAS-LRS can identify single nucleotide variants with nearly the same accuracy as the short-read platform, and elucidate complex structural variations including mobile element insertions. In addition, we showed that off-target reads can be used to genotype common SNPs genome-wide. Furthermore, it is possible to identify allele-specific methylation aberrations. Thus, a single platform can simultaneously capture the genomic and epigenetic status of monogenic disease-causing genes as well as polygenic effects^56^.

One of the major challenges for LRS at present is the accurate detection of Indels, as in other studies using error-prone long reads. It may require some heuristics such as devising effective post-filtering of Indels which are located in homopolymers. At the same time, measurement technology has continued to make great progress. In fact, the evaluation in this study was mostly performed using the R9.4.1 flow cell, which is a slightly earlier generation, and the accuracy of Indels detection would increase if a newer sequencing kit (Kit V14) were used^57^. In addition, recent ONT duplex technologies, which attach adapters to both strands of a DNA molecule and sequence them from both sides, have been reported to achieve Q30 accuracy. We are optimistic that the problem of LRS Indels detection problem of LRS will be resolved in the near future.

Mobile element insertions such as SVA are notoriously difficult to detect with existing short-read sequencing platforms. In two FAP patients, we successfully identified SVA insertions and their detailed characteristics such as their sites of origin. In fact, the detection of mobile element insertions may contribute to future therapeutic options as well as diagnostics. A groundbreaking study administered a personalized antisense oligonucleotide therapy for a child with Batten disease that targets abnormal splicing caused by a sporadic SVA insertion^58^. Although a current definitive treatment for FAP patients is prophylactic colectomy before colorectal cancer develops, it has many limitations such as surgical complications and decreased quality of life. Tailor-made medicine such as an antisense oligonucleotide by examining the genomes of FAP patients and other hereditary tumor-related diseases may be an alternative treatment in the future.

We have demonstrated that genotyping of common SNPs can be performed accurately using off-target reads. In this study, we focused on genotyping SNPs with relatively high minor allele frequencies (MAFs). The accuracy of genotyping at rarer variants will continue to improve in the future as large-scale biobank data accumulate and reference panels become more well-stocked, which may even allow for the identification of pathogenic variants with intermediate MAF despite those being located in the off-target region. An increase in concentration ratio due to improvement in sequencing accuracy and real-time alignment computational time may reduce off-target reads for genome-wide genotyping. In such cases, we will be able to explicitly adjust settings, such as specifying a time or the number of pores in which adaptive sampling is performed.

The high genotyping quality of GLIMPSE suggests that haplotype phasing could potentially be accomplished even with low coverage, by leveraging the population’s linkage disequilibrium information. However, it is important to note that the population linkage disequilibrium-based approach has inherent challenges, such as the inability to phase rare variants. In the future, a method integrating both variant co-occurrence information in long reads and population linkage disequilibrium data will likely be essential.

Allele-specific methylation status could be assessed without the need for experiments such as bisulfite conversion. The current study focused on target regions of known cancer predisposition genes. However, as a previous report demonstrated that methylation information from low-coverage sequencing can reveal cancer subtypes^59^, there is a possibility that information from off-target regions can be effectively used in the future.

In this study, we focused on identifying variants in the germline genome. On the other hand, somatic mutations are more difficult to detect than germline mutations due to the impurity of the tumor, the presence of subclones, and the low frequency of variant alleles. However, it is expected that the accuracy of sequencing and the amplification rate of adaptive sampling will increase in the future due to the development of products such as flow cells and reagents and the improvement of various analytical instruments. We are optimistic that reliable somatic mutation detection, including Indels, will eventually be achievable in the near future, and that TAS-LRS will replace the current panel sequence based on a short-read platform.

In summary, we showed that our workflow enables SNVs/Indels detection, methylation status detection, and genome-wide common SNP genotyping. Moreover, our workflow is not just long-read target sequencing to cover the disadvantages of short-read sequencing, but also SNP array sequencing of whole genomic regions and haplotype-by-haplotype methylation analysis of target regions without additional operations. This striking method can be flexibly executed by simply specifying the target regions computationally.

## Methods

### Ethics approval and consent to participate

The research protocol was approved by the Ethics Committee of the National Cancer Center Hospital (Tokyo, Japan) (approval #2013-303). Written informed consent for clinical genetic testing and genomic analysis was obtained from patients. All patient information was deidentified. We have complied with all relevant ethical regulations including the Declaration of Helsinki.

### Adaptive sampling sequencing with GridION

DNA was obtained after the isolation of plasma from blood samples. For each sample, 10 μg of genomic DNA was sheared using a Covaris g-TUBE by centrifugation at 4200 rpm for 60 seconds, followed by inversion and centrifugation again at 4200 rpm for 60 seconds. DNA for sequencing was prepared using the ONT Ligation Kit (SQK-LSK110 or SQK-LSK114) according to the manufacturer’s instructions. Each library was loaded mostly on R9.4.1 FLO-MIN106D (29 patients) and rarely on R10.4 FLO-MIN112 (4 patients) flow cells. Sequencing was performed in “hac” mode using the adaptive sampling option for 72 hours with two additional library loadings once every 24 hours after nuclease flushing of a flow cell using the Flow Cell Wash Kit (EXP-WSH004).

### Alignment of the sequencing data

FAST5 files were base-called using the high-accuracy model in Guppy (ver.6.0.7). All FASTQ files were aligned using Minimap2 (ver.2.22-r1101)^60^ with the “-ax map-ont -t 8 -p 0.1–MD“ option to the GRCh38 human reference genome, and converted into BAM files, and sorted and indexed with SAMtools (ver.1.13)^61^.

### Detection, annotation, and prioritization of SNVs/Indels

Denovo SNVs, Indels detection were performed using PEPPER-Margin-DeepVariant (ver.0.8.0)^7^, via “run_pepper_margin_deepvariant call_variant” command with the “–ont_r9_guppy5_sup” mode, and filtered out the SNVs/Indels with <= 10 quality values by the software. We focused on the SNVs/Indels of the region where the maximum regions of all corresponding transcripts are registered in the GENCODE Basic gene annotation release 38, excluding the region of simple repeat regions and segmental duplication regions. Then, using Ensembl Variant Effect Predictor (VEP) ver.105.0^62^ and our in-house custom scripts, we annotated SNVs/Indels with the deleteriousness prediction (CADD ver.1.6^63^, ClinVar^33^, and LOFTEE^34^), the splicing prediction (SpliceAI^64^), splicing variant database (SAVNet^65^ and IRAVDB^66^), and the population allele frequencies (gnomAD ver.3.1.2^34^ and ToMMo 14JPN^38^).

To select candidates of pathogenic SNVs/Indels, we first removed those commonly found in general populations (allele frequencies equal to or greater than 0.01 by gnomAD v.3.1.2 and ToMMo 38KJPN)^34,67,68^. Then, variants satisfying either of the following conditions were extracted as putative pathogenic variants.

1. Annotated as “Pathogenic“ or “Likely pathogenic“ by ClinVar^33^.
2. Affecting essential splice-site (GT-AG) or those whose delta score by SpliceAI^64^ is 0.50 or greater.
3. Loss of function variants and deemed as “High-confidence (HC)” by LOFTEE^34^.

### Detection, annotation, and prioritization of SVs

SVs were detected using nanomonsv (ver.0.7.0)^69^. After “nanomonsv parse” was performed for each BAM file, we executed “nanomonsv get” with “–single_bnd–use_racon” option to identify single breakend SVs. We also included a dummy matched control and the control panel data created from the sequencing data from Human Pangenome Reference Consortium (provided by Zenodo (https://zenodo.org/record/7017953#.Y8uP-uxByZw). For the dummy match control, we used whole-genome long-read sequencing data (by Oxford Nanopore Technologies, base-called with Guppy 5.0.11) of a Japanese male (NA18989) provided by the Human Genome Structural Variation Consortium^70,71^. Then, we filtered out SVs that satisfy the following conditions (false-positive SV elimination filter):

1. One or both of the SV breakpoints are present in the unplaced contig, decoy sequence.
2. Neither SV breakpoint is located within the target region.
3. Deletion, insertion, and tandem duplication type SVs are included in the simple repeat region (including 10 bp margin).

Furthermore, the following categories of SVs were extracted as putative pathogenic SVs (putative pathogenic SVs extraction):

1. Deletions involving the coding regions of the 147 target genes.
2. Duplications that alter the coding sequences of 147 genes. More specifically, those that have at least one breakpoint within the target genes excluding untranslated regions and the amplified regions span the coding sequences.
3. Inversions or translocations that disrupt the coding sequences of 147 genes. That is, those that have at least one of the breakpoints in a gene region excluding the untranslated regions.

We also included single breakend SVs detected by nanomonsv with the “–single_bnd–use_racon” options.

### Whole genome short read sequencing analysis

We performed alignment of the FASTQ file to the GRCh38 human reference genome using BWA-MEM version 0.7.15^72^ with the options “-Y -T 0 -K 10000000”. We then sorted the files and marked duplicates using the GATK (version 4.1.0.0) SortSam and MarkDuplicates commands, respectively. Subsequently, we converted the file format from BAM to CRAM using SAMtools version 1.9.

For SNVs and Indels calling, we used GATK HaplotypeCaller with the option “-ERC GVCF.” Then, joint-calling was performed with GATK GenomicsDBImport and GATK GenotypeGVCF commands. For SNVs, after selecting SNP with the GATK SelectVariants command and “-select-type SNP,” we performed variant filtering with GATK VariantFiltration with the filtering option “QD < 2.0”–filter-name “QD2” -filter “QUAL < 30.0”–filter-name “QUAL30” -filter “SOR > 3.0”–filter-name “SOR3” -filter “FS > 60.0”–filter-name “FS60” -filter “MQ < 40.0”–filter-name “MQ40” -filter “MQRankSum < -12.5”–filter-name “MQRankSum-12.5” -filter “ReadPosRankSum < -8.0”–filter-name “ReadPosRankSum-8.” For Indels, after GATK SelectVariants–select-type INDEL, we performed filtering with the filtering option of “QD < 2.0”–filter-name “QD2” -filter “QUAL < 30.0”–filter-name “QUAL30” -filter “FS > 200.0”–filter-name “FS200” -filter “ReadPosRankSum < −20.0”–filter-name “ReadPosRankSum-20.”

### Whole transcriptome analysis for evaluating SVA insertions on *APC* gene

For each sample, FASTQ files were aligned to the GRCh38 human reference genome as performed previously^66^ but using STAR version 2.7.9a^73^. Subsequently, BAM files were sorted and indexed using SAMtools. For regions with SVA insertions, sashimi plots were generated using ggsashimi version 1.1.5^74^.

### Common SNP genotype calling using GLIMPSE

First, we generated a reference panel VCF file based on the joint-called VCF file of 8570 short-read platform whole genome sequencing data collected from the National Center Biobank Network (NCBN) as well as 3202 sequencing data from 1000 Genomes Project generated in the previous study^52^. We retained only single nucleotide variants that met the following criteria: a *p*-value in the Hardy–Weinberg Equilibrium goodness-of-fit (HWE) of ≥0.0001, a missing genotype ratio F_MISSING of ≤0.05, and a total number of allele counts (AC) of ≥5. Then, for the filtered joint-called VCF, we performed haplotype phasing using Beagle 5.2^75^ with the genetic map obtained from Beagle website (https://bochet.gcc.biostat.washington.edu/beagle/genetic_maps/plink.GRCh38.map.zip).

Then, we performed genotyping for each BAM file from TAS-LRS using GLIMPSE (ver.1.1.1)^28^ using the above reference panel. First, multiallelic sites of VCF files were split into biallelic records using the “bcftools norm -m -any” command, then converted to tsv files using “bcftools query -f’%CHROM\t%POS \t%REF,%ALT\n’ ${REFVCF} | bgzip -c” command and indexed the tsv file using “tabix -s1 -b2 -e2” command. In addition to the options described in the tutorial, genotype likelihoods for a single individual at specific positions were computed using bcftools mpileup with the “-X ont” option and bcftools call with the “-P 0.01” option. The GLIMPSE_chunk command to generate imputation regions for each chromosome was performed with the “–window-size 2000000” and “–buffer-size 200000” options. The GLIMPSE_phase command to impute and phase a whole chromosome was run using default parameters. Fine-scale genetic maps were downloaded from the URL　https://zenodo.org/record/4078748. The GLIMPSE_ligate and the GLIMPE_sample commands were performed as default parameters.

To compare genotyping results between TAS-LRS using GLIMPSE and WG-SRS using GATK, we excluded simple repeat regions, segmental duplication regions, and regions to which alternative haplotype sequences match, which we downloaded from the annotation database for the UCSC Genome Browser (https://hgdownload.soe.ucsc.edu/goldenPath/hg38/database). We used GLIMPSE2_concordance^76^ tool to evaluate the accuracy of genotyping. We followed the GLIMPSE2 tutorial (https://odelaneau.github.io/GLIMPSE/docs/tutorials/getting_started/) and checked the imputation accuracy using the “GLIMPSE2_concordance” command with the “–min-val-dp 8–min-val-gl 0.9999–bins 0.000 0.001 0.002 0.005 0.010 0.050 0.100 0.200 0.500” options.

### Allele-specific methylation analysis

First, we classified each read in the BAM file using WhatsHap (ver.1.4)^77^ (“whatshap haplotag” command was used to tag reads by haplotype with “–skip-missing-contigs” and “–ignore-read-groups” options) based on the phased common SNP genotype VCF file obtained using GLIMPSE in the previous subsection. f5c (ver.1.2)^78^ was then used for the methylation calling. After indexing the FASTA file, the command “f5c call-methylation” was executed. Next, the output file containing the log-likelihood ratio of methylation per read and CG dinucleotide was divided into three files (“haplotype 1”, “haplotype 2”, and “haplotype unclassified”). Finally, the allele-specific methylation frequencies for each CG dinucleotide were calculated using “meth-freq” command on the above split files.

First, at each CpG site, we set the methylation ratio as the number of methylated bases divided by the sum of methylated and non-methylated bases (#methylated bases/(#methylated bases + #non-methylated bases)). Next, we define CpG sites with a methylation ratio of 0–0.2 in all 10 samples as “normally unmethylated CpG sites”, and CpG sites with a methylation ratio of 0.8–1 as “normally methylated CpG sites”. For the analysis of each target sample, if the methylation ratio at normally unmethylated CpG sites is above 0.2 and the *p*-value from the two-sided Fisher’s exact test (comparing the total of #methylated bases and #non-methylated bases in the control samples, and those of the target sample) is 0.05 or below, it is identified as hypermethylation. Additionally, at normally methylated sites, if the methylation ratio in the target sample is 0.8 or below and the two-sided Fisher’s exact test *p*-value is 0.05 or below, it is identified as hypomethylation.

Finally, to identify aberrantly methylated genes, we counted the number of CpGs situated 2000 bp upstream from the transcription start site that showed abnormal methylation (as defined by either hypomethylation or hypermethylation) for each target gene. We then pinpointed genes where either the average −log10(*p*-value) across the abnormally methylated CpG sites was 4.5 or higher, or the count of such sites reached 10 or more.

To convert the BAM file for methylation visualization via Integrative Genomic Viewer^68^, we used the convert_bam_for_methylation.py script (https://github.com/timplab/nanopore-methylation-utilities)^79^.

### The comparison of polygenic risk score

PRS was calculated using PLINK (ver.1.90 beta)^80^ according to the tutorial page (https://choishingwan.github.io/PRS-Tutorial/plink/)^81^. We used GWAS summary statistics^53^ for 12 cancer types (breast cancer, biliary tract cancer, cervical cancer, colorectal cancer, endometrial cancer, esophageal cancer, gastric cancer, hepatocellular cancer, lung cancer, ovarian cancer, pancreatic cancer, and prostate cancer) downloaded from Biobank Japan (http://jenger.riken.jp/result) as base data. We converted the coordinates of these summary statistics to hg38 using the UCSC LiftOver^82^ tool and processed them according to the tutorial.

For the target data, we used VCF files for common SNPs by GLIMPSE for TAS-LRS merged across samples. We also used a phased VCF file for the corresponding individuals extracted from the reference panel produced by BEAGLE for WG-SRS for corresponding individuals. For the QC of the target data, we mostly followed the tutorial page. However, we modified the option of–indep-pairwise to 50000 5000 0.50 when removing highly correlated SNPs. Also, we did not perform sample exclusion by the standard deviation of the *F* coefficient due to the small sample size. Finally, we performed clumping and calculated PRS for each cancer. Then, we compared the PRS calculated by TAS-LRS and WG-SRS for each individual.

### Dimension reduction with principal components analysis

Principal components analysis (PCA) was performed using PLINK (ver.1.90 beta)^80^.

First, the linkage pruning was performed by PLINK with the option of–indep-pairwise 50 10 0.10–geno 0.01–maf 0.1 –freq.” Then, we next performed PLINK with “–pca” to obtain eigenvector and values.

### Reporting summary

Further information on research design is available in the Nature Research Reporting Summary linked to this article.

### Supplementary information

REPORTING SUMMARY

Supplementary Information

Supplementary Data 1

Supplementary Data 2

Supplementary Data 3

Supplementary Data 4

Supplementary Data 5

Supplementary Data 6

## Data Availability

The raw nanopore sequence data via target adaptive sampling used in this study are deposited in the National Bioscience Database Center (NBDC) Human Database and are available at the Japanese Genotype–phenotype Archive (JGA) with accession codes JGAS000628.

## Code availability

The workflow used in this study is available on GitHub at https://github.com/ncc-gap/ASWorkflow.
