# Supplementary material for: Assessing the efficacy of target adaptive sampling long-read sequencing through hereditary cancer patient genomes
Source: NPJ Genom Med. 2024 Feb 17;9:11. doi: 10.1038/s41525-024-00394-z (PMC10874402; doi:10.1038/s41525-024-00394-z)
Supplement: Supplementary file 1 — REPORTING SUMMARY [file 41525_2024_394_MOESM1_ESM.pdf]

nature portfolio

Corresponding author(s): Yuichi Shiraishi

Last updated by author(s): Oct 19, 2023

Reporting Summary

Nature Portfolio wishes to improve the reproducibility of the work that we publish. This form provides structure for consistency and transparency in reporting. For further information on Nature Portfolio policies, see our Editorial Policies and the Editorial Policy Checklist.

Statistics

For all statistical analyses, confirm that the following items are present in the figure legend, table legend, main text, or Methods section.

n/a	Confirmed	
☐	☒	The exact sample size (*n*) for each experimental group/condition, given as a discrete number and unit of measurement
☐	☒	A statement on whether measurements were taken from distinct samples or whether the same sample was measured repeatedly
☐	☒	The statistical test(s) used AND whether they are one- or two-sided<br>*Only common tests should be described solely by name; describe more complex techniques in the Methods section.*
☒	☐	A description of all covariates tested
☒	☐	A description of any assumptions or corrections, such as tests of normality and adjustment for multiple comparisons
☐	☒	A full description of the statistical parameters including central tendency (e.g. means) or other basic estimates (e.g. regression coefficient) AND variation (e.g. standard deviation) or associated estimates of uncertainty (e.g. confidence intervals)
☐	☒	For null hypothesis testing, the test statistic (e.g. *F*, *t*, *r*) with confidence intervals, effect sizes, degrees of freedom and *P* value noted<br>*Give P values as exact values whenever suitable.*
☒	☐	For Bayesian analysis, information on the choice of priors and Markov chain Monte Carlo settings
☒	☐	For hierarchical and complex designs, identification of the appropriate level for tests and full reporting of outcomes
☒	☐	Estimates of effect sizes (e.g. Cohen's *d*, Pearson's *r*), indicating how they were calculated

Our web collection on statistics for biologists contains articles on many of the points above.

Software and code

Policy information about availability of computer code

Data collection	Data collection methods are described in the Methods section.<br><br>Adaptive sampling sequencing with GridION<br>DNA was obtained after the isolation of plasma from blood samples. For each sample, 10 μg of genomic DNA was sheared using a Covaris g-TUBE by centrifugation at 4,200 rpm for 60 seconds, followed by inversion and centrifugation again at 4,200 rpm for 60 seconds. DNA for sequencing was prepared using the ONT Ligation Kit (SQK-LSK110) according to the manufacturer's instructions. Each library was loaded mostly on R9.4.1 FLO-MIN106D (29 patients) and rarely on R10.4 FLO-MIN112 (4 patients) flow cells. Sequencing was performed in “hac” mode using the adaptive sampling option for 72 hours with two additional library loadings once every 24 hours after nuclease flushing of a flow cell using the Flow Cell Wash Kit (EXP-WSH004).<br><br>Detection, annotation, and prioritization of SVs<br>For the dummy match control, we used whole-genome long-read sequencing data (by Oxford Nanopore Technologies, base-called with Guppy 5.0.11) of a Japanese male (NA18989) provided by the Human Genome Structural Variation Consortium.
Data analysis	Data analysis methods are described in the Methods section.<br><br>Alignment of the sequencing data<br>Guppy (ver.6.0.7),Minimap2 (ver.2.22-r1101) , Samtools (ver.1.13)<br><br>Detection, annotation, and prioritization of SNVs/Indels<br>PEPPER-Margin-DeepVariant (ver.0.8.0), Ensembl Variant Effect Predictor (ver.105.0)

Detection, annotation, and prioritization of SVs
nanomonsv (ver.0.7.0)

Whole genome short read sequencing analysis
BWA-MEM (ver.0.7.15), GATK (ver.4.1.0.0), Samtools (ver.1.9).

Whole transcriptome analysis for evaluating SVA insertions on APC gene
STAR (ver.2.7.9a), Samtools (ver.1.9), ggsashimi (ver.1.1.5).

Common SNP genotype calling using GLIMPSE
GLIMPSE (ver.1.1.1), WhatsHap (ver.1.4), f5c (ver.1.2)

The comparison of polygenic risk score
PLINK (ver.1.90 beta)

Dimension reduction with principal components analysis
PLINK (ver.1.90 beta)

For manuscripts utilizing custom algorithms or software that are central to the research but not yet described in published literature, software must be made available to editors and reviewers. We strongly encourage code deposition in a community repository (e.g. GitHub). See the Nature Portfolio guidelines for submitting code & software for further information.

Data

Policy information about availability of data

All manuscripts must include a data availability statement. This statement should provide the following information, where applicable:

- Accession codes, unique identifiers, or web links for publicly available datasets
- A description of any restrictions on data availability
- For clinical datasets or third party data, please ensure that the statement adheres to our policy

Data availability statement is described in the Data Availability section.
The workflow used in this study is available on GitHub at https://github.com/ncc-gap/ASWorkflow. The raw nanopore sequence data via target adaptive sampling used in this study will be available through the public sequence repository service.

Research involving human participants, their data, or biological material

Policy information about studies with human participants or human data. See also policy information about sex, gender (identity/presentation), and sexual orientation and race, ethnicity and racism.

Reporting on sex and gender	Although sex information is collected in patient registration, we did not use sex information in our analysis.
Reporting on race, ethnicity, or other socially relevant groupings	As principal component analysis (PCA) of the genotype data indicated that one in thirty-three patients was likely to be of different ancestry, we performed race ascertainment based on the information collected.
Population characteristics	We enrolled 33 genomes collected from suspected hereditary cancer patients.
Recruitment	We recruited samples enrolled in a research protocol approved by the Ethics Committee of the National Cancer Center Hospital (Tokyo) (approval number 2013-303).
Ethics oversight	Ethical oversight was described in the Ethical Approval and Consent to Participate section.<br><br>The research protocol was approved by the Ethics Committee of the National Cancer Center Hospital (Tokyo, Japan) (approval #2013-303). Written informed consent for clinical genetic testing and genomic analysis was obtained from patients. All patient information was deidentified.

Note that full information on the approval of the study protocol must also be provided in the manuscript.

Field-specific reporting

Please select the one below that is the best fit for your research. If you are not sure, read the appropriate sections before making your selection.

☒ Life sciences ☐ Behavioural & social sciences ☐ Ecological, evolutionary & environmental sciences

For a reference copy of the document with all sections, see nature.com/documents/nr-reporting-summary-flat.pdf

Life sciences study design

All studies must disclose on these points even when the disclosure is negative.

Sample size	Sample size was determined by sample availability.
Data exclusions	No data were excluded from the analysis.
Replication	We compared the detection between TAS-LRS and high-coverage whole-genome short-read sequence (WG-SRS) for 22 cases where matched WG-SRS data were available to evaluate the accuracy of the detection of SNVs/Indels.<br>We assessed genotyping accuracy using low-coverage ONT sequencing data with the widely recognized HG001 sample, employing down-sampling. Whole transcriptome analysis was performed on two cases in which SVA insertion into the APC gene was suspected, and splicing abnormalities were confirmed. In one case of MLH1 epimutation, we added immunohistochemistry to confirm MLH1 loss.
Randomization	Samples were not randomized.
Blinding	Investigators were not blinded.

Reporting for specific materials, systems and methods

We require information from authors about some types of materials, experimental systems and methods used in many studies. Here, indicate whether each material, system or method listed is relevant to your study. If you are not sure if a list item applies to your research, read the appropriate section before selecting a response.

Materials & experimental systems

n/a	Involved in the study	
☒	☐	Antibodies
☒	☐	Eukaryotic cell lines
☒	☐	Palaeontology and archaeology
☒	☐	Animals and other organisms
☒	☐	Clinical data
☒	☐	Dual use research of concern
☒	☐	Plants

Methods

n/a	Involved in the study	
☒	☐	ChIP-seq
☒	☐	Flow cytometry
☒	☐	MRI-based neuroimaging

Plants

Seed stocks	*Report on the source of all seed stocks or other plant material used. If applicable, state the seed stock centre and catalogue number. If plant specimens were collected from the field, describe the collection location, date and sampling procedures.*
Novel plant genotypes	*Describe the methods by which all novel plant genotypes were produced. This includes those generated by transgenic approaches, gene editing, chemical/radiation-based mutagenesis and hybridization. For transgenic lines, describe the transformation method, the number of independent lines analyzed and the generation upon which experiments were performed. For gene-edited lines, describe the editor used, the endogenous sequence targeted for editing, the targeting guide RNA sequence (if applicable) and how the editor was applied.*
Authentication	*Describe any authentication procedures for each seed stock used or novel genotype generated. Describe any experiments used to assess the effect of a mutation and, where applicable, how potential secondary effects (e.g. second site T-DNA insertions, mosiacism, off-target gene editing) were examined.*
