# Supplementary material for: Assessing the efficacy of target adaptive sampling long-read sequencing through hereditary cancer patient genomes
Source: NPJ Genom Med. 2024 Feb 17;9:11. doi: 10.1038/s41525-024-00394-z (PMC10874402; doi:10.1038/s41525-024-00394-z)
Supplement: Supplementary file 2 — Supplementary Information [file 41525_2024_394_MOESM2_ESM.pdf]

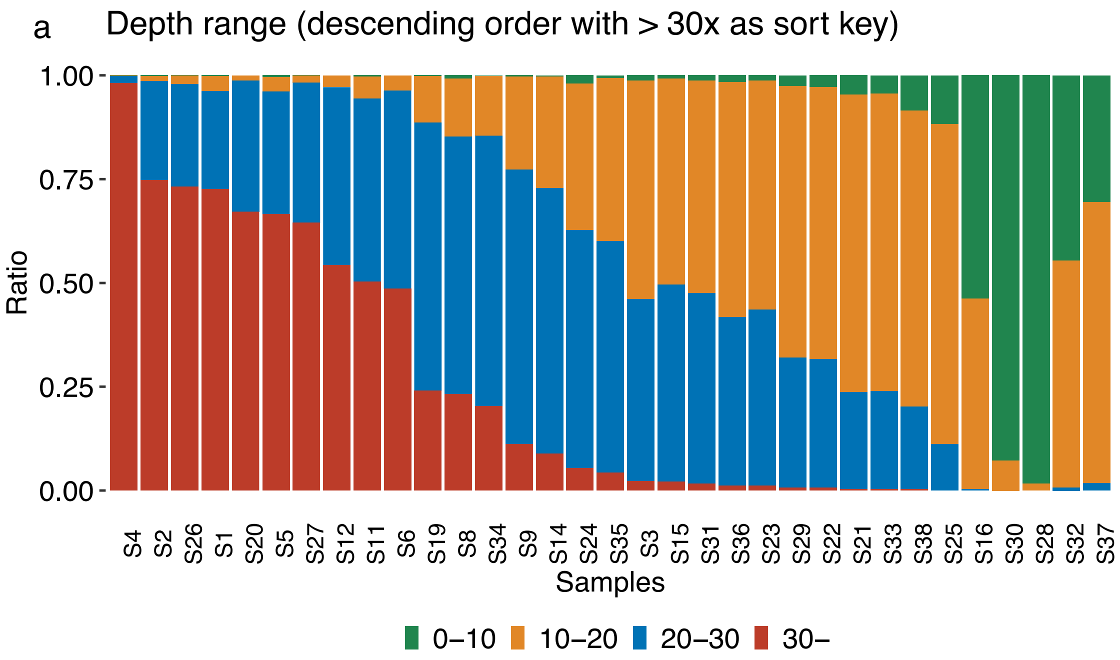

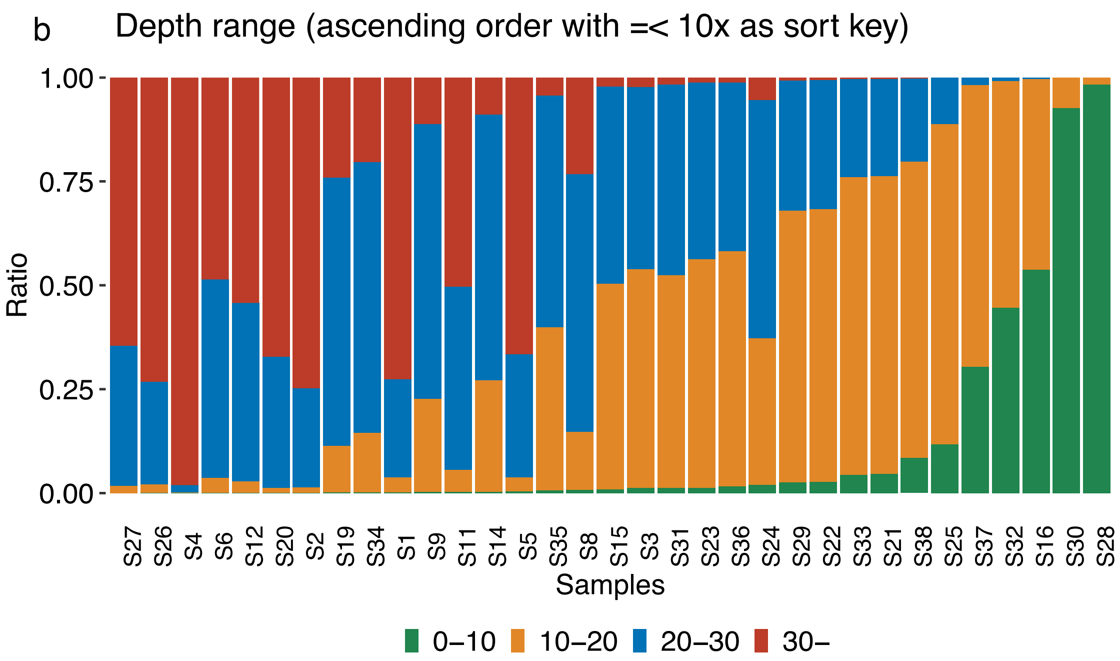

Supplementary Figure 1: Summary of sequence coverage of TAS-LRS sequencing data. The barplot shows the percentage of each sequence coverage range ([0, 10], [10, 20], [20, 30], [30, ∞] in the target area (a) in descending order with [30, ∞], and (b) in ascending order with [0, 10] as the sort keys.

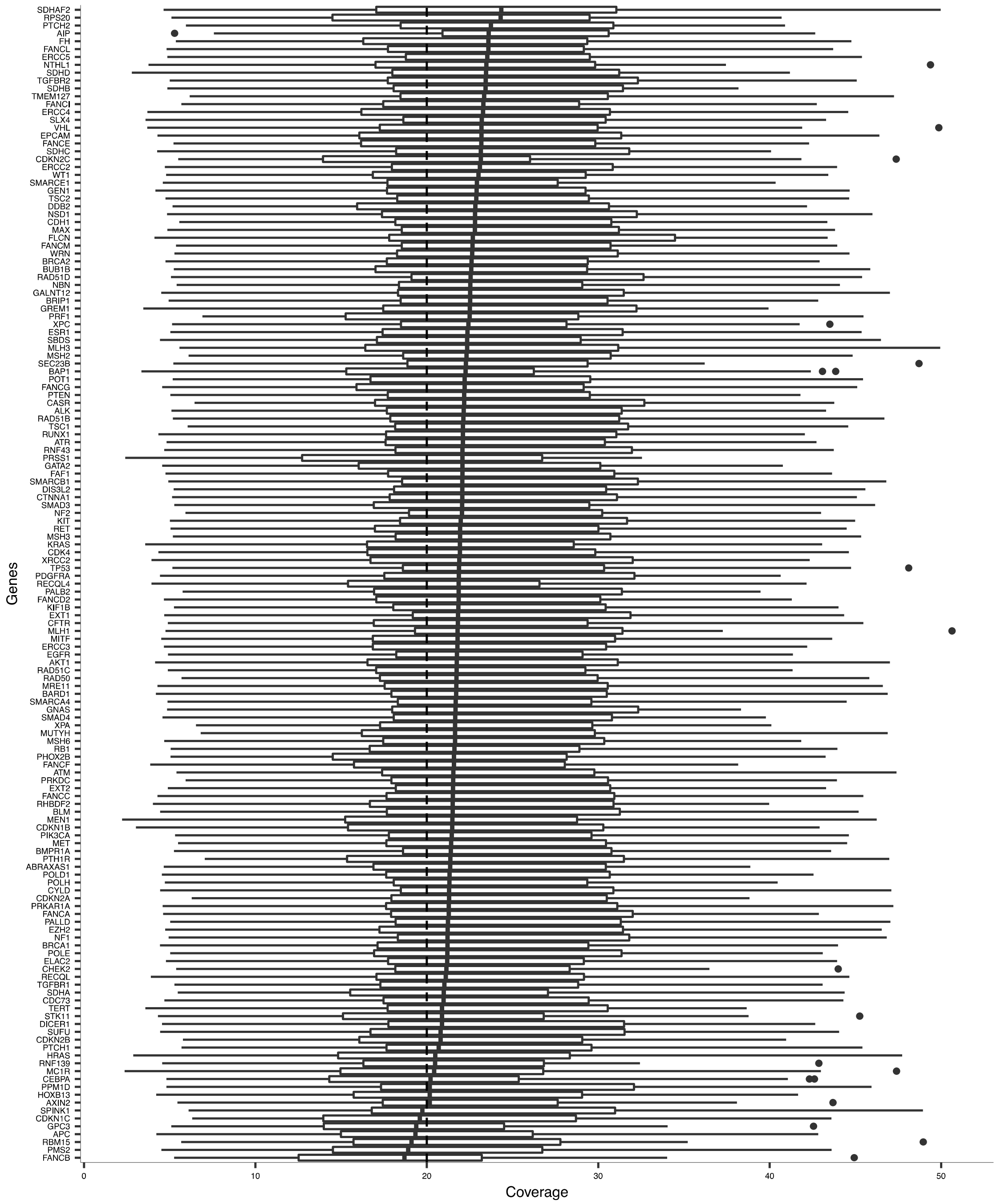

Supplementary Figure 2: Gene-wise sequence coverage of TAS-LRS sequencing data. Box plots show medians (lines), interquartile ranges (IQRs; boxes), ±1.5 × IQRs (whiskers), and outliers (dots).

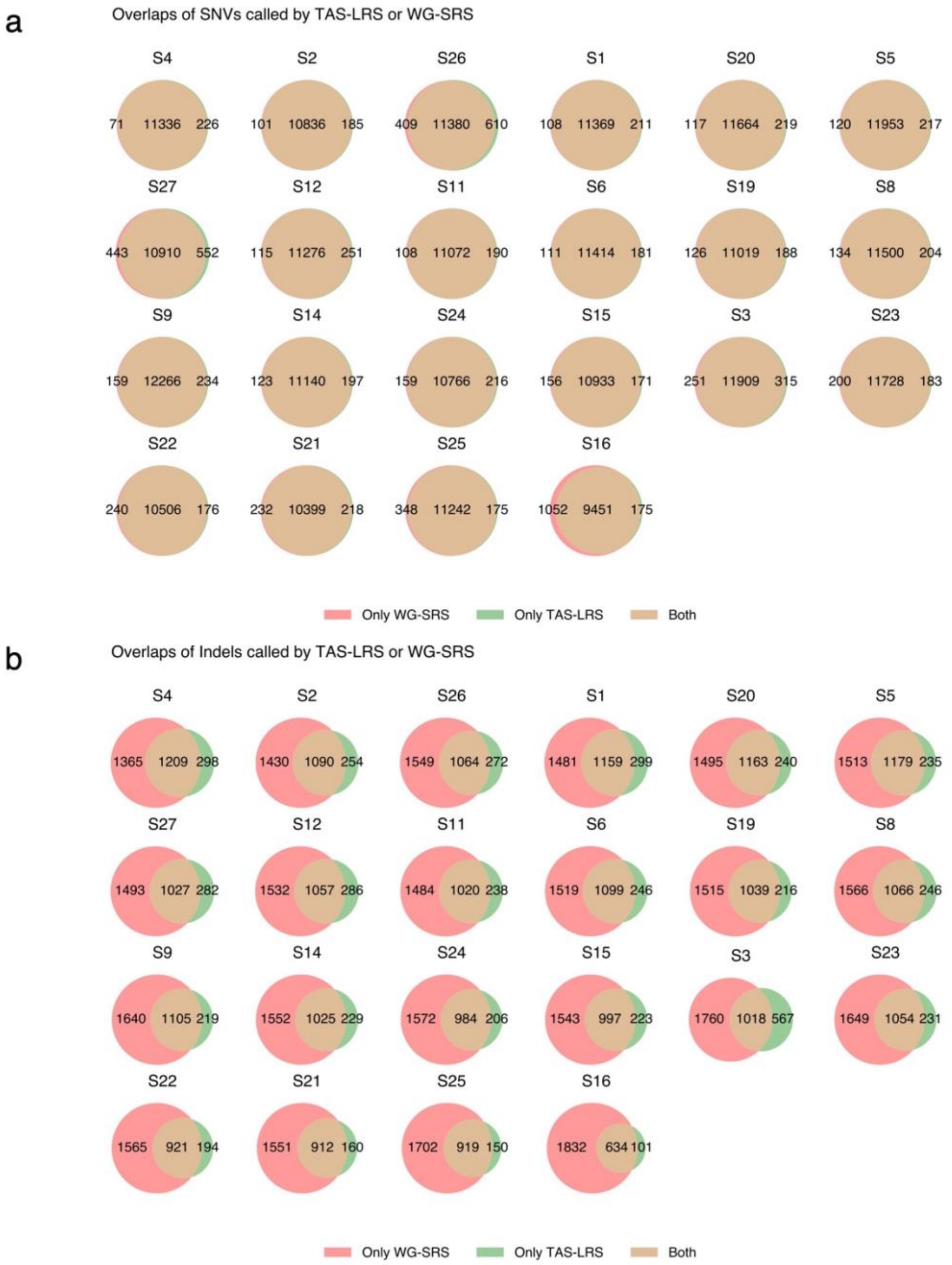

Supplementary Figure 3: Venn diagrams of SNVs/Indels by TAS-LRS and WG-SRS for each sample. Venn diagrams are sorted in descending order with mean sequence coverage in the target regions.

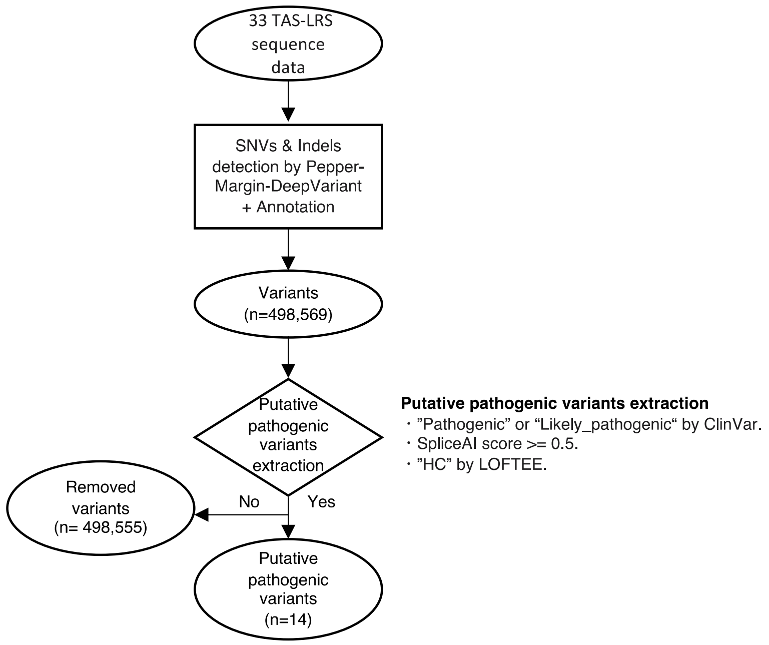

Supplementary Figure 4: Flowchart for putative pathogenic SNVs/indels identification.

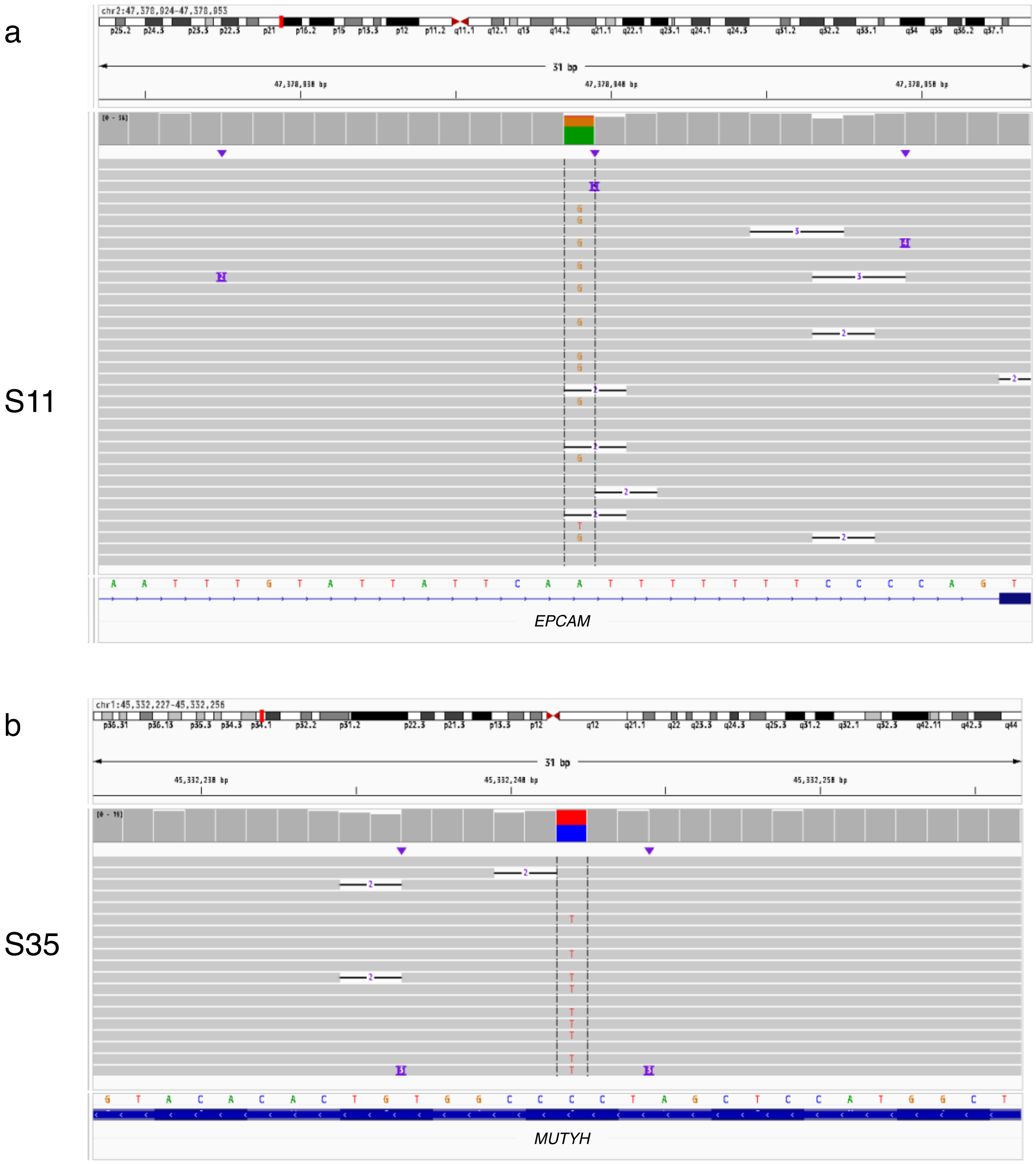

Supplementary Figure 5: Two candidate pathogenic variants. (a,b) Alignment view of the variant (a) in the *EPCAM* gene of the patient S11 and (b) in the *MUTYH* gene of the patient S35.

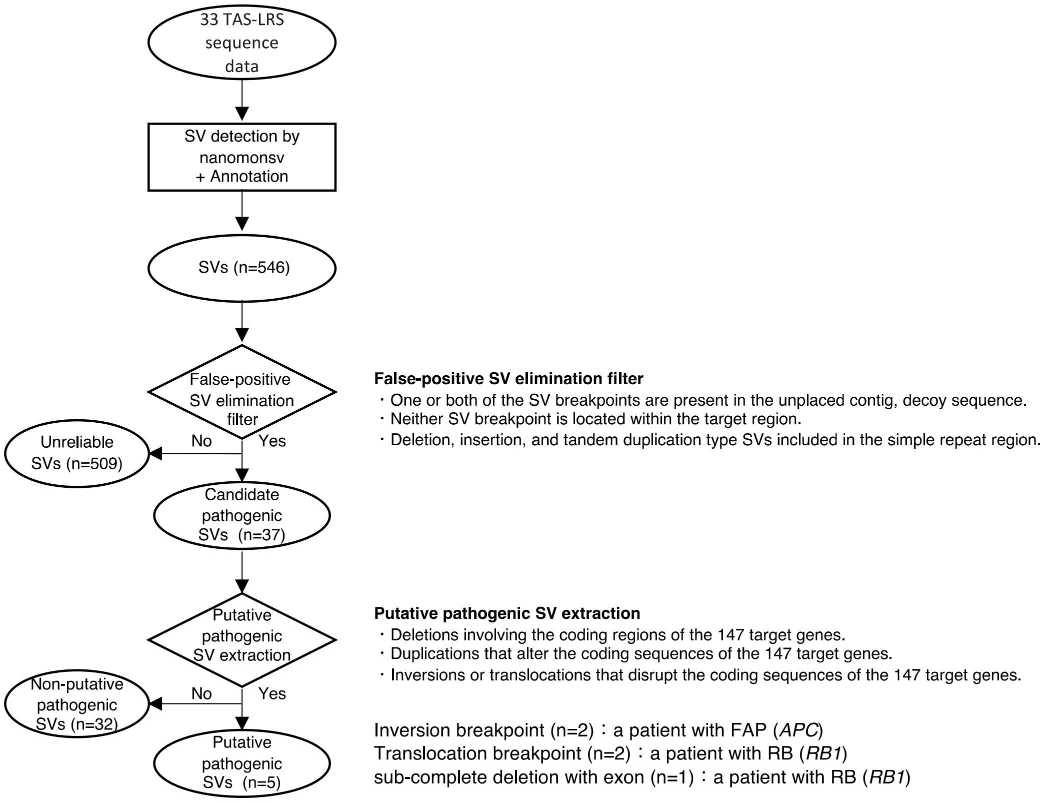

Supplementary Figure 6: Flowchart for putative pathogenic SV detection for nanomonsv canonical SV module.

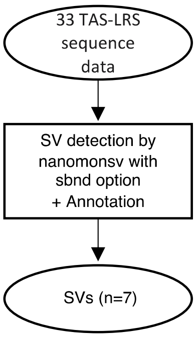

Supplementary Figure 7: Flowchart for putative pathogenic SV detection for nanomonsv single-breakend SV module.

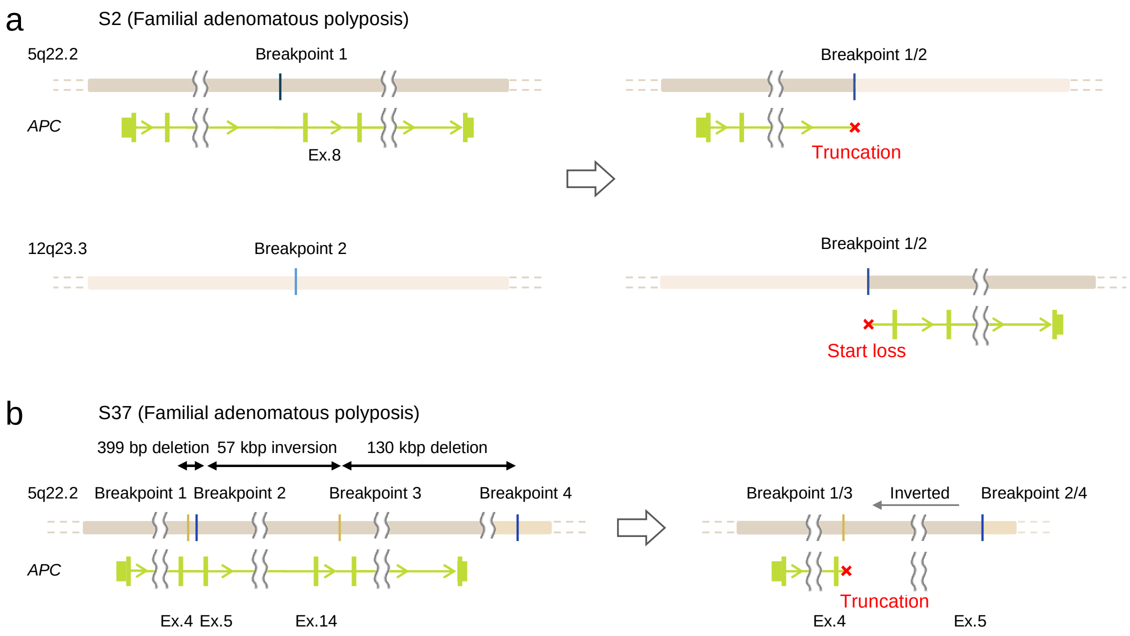

Supplementary Figure 8: Schematic representation of structural variations of the *APC* gene in two patients with familial adenomatous polyposis. (a) DNA swapping occurred at two breakpoints in the 7th intron of the *APC* gene and in the intergenic region at 12q23.3, forming a balanced translocation. (b) Reciprocal inversions occurred at breakpoints in the 4th and 14th intron of the *APC* gene, involving the deletions of 399 bp and 130 kbp.

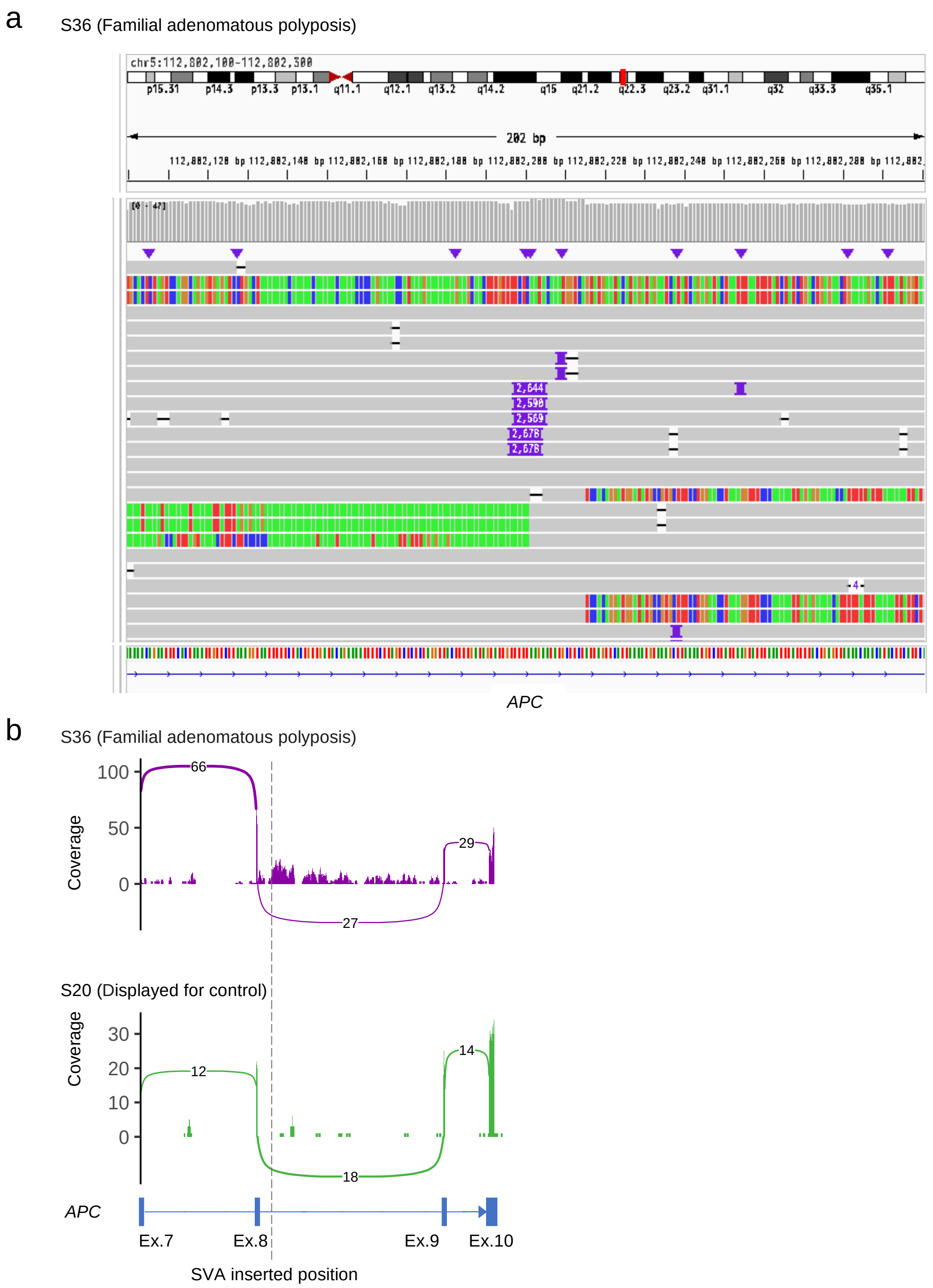

Supplementary Figure 9: Details of SVA-derived insertion into the intronic region of the *APC* gene in a patient with familial adenomatous polyposis. (a) The IGV displayed long-read sequencing data and transcript sequencing data showing an SVA-derived insertion of 2,678 bp in the 8th intron of the *APC* gene. (b) The whole transcriptome sequence showed specific intron retention at the near exon-intron boundary.

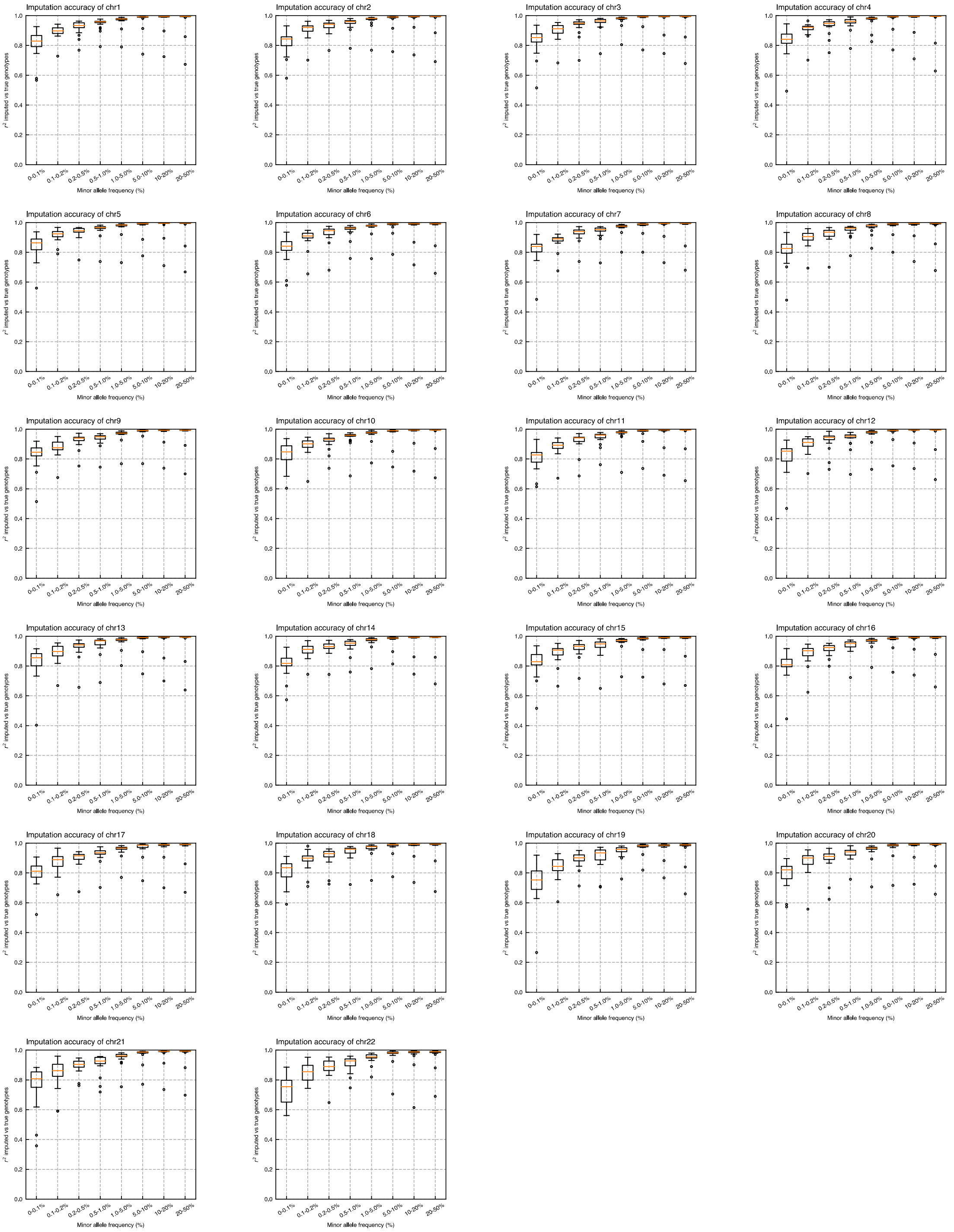

Supplementary Figure 10: Imputation accuracy of TAS-LRS measured for each chromosome and each minor allele frequency range. Genotyping by WG-SRS was used as the golden standard. Box plots show medians (lines), interquartile ranges (IQRs; boxes), ±1.5 × IQRs (whiskers), and outliers (dots).

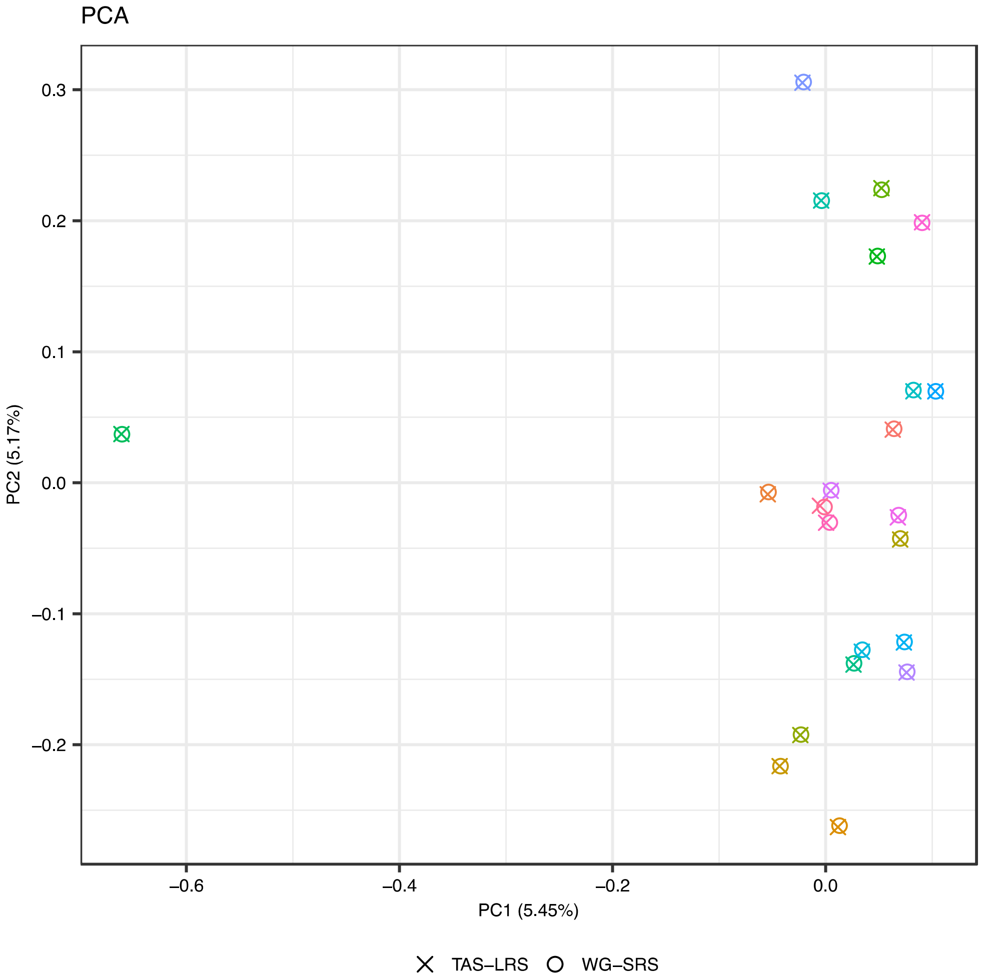

Supplementary Figure 11: **Principal component analysis (PCA) of genotype results from both TAS-LRS and WG-SRS for each individual (distinguished by color).** This figure includes one outlier sample probably originating from different ancestries. See also Figure 6b.

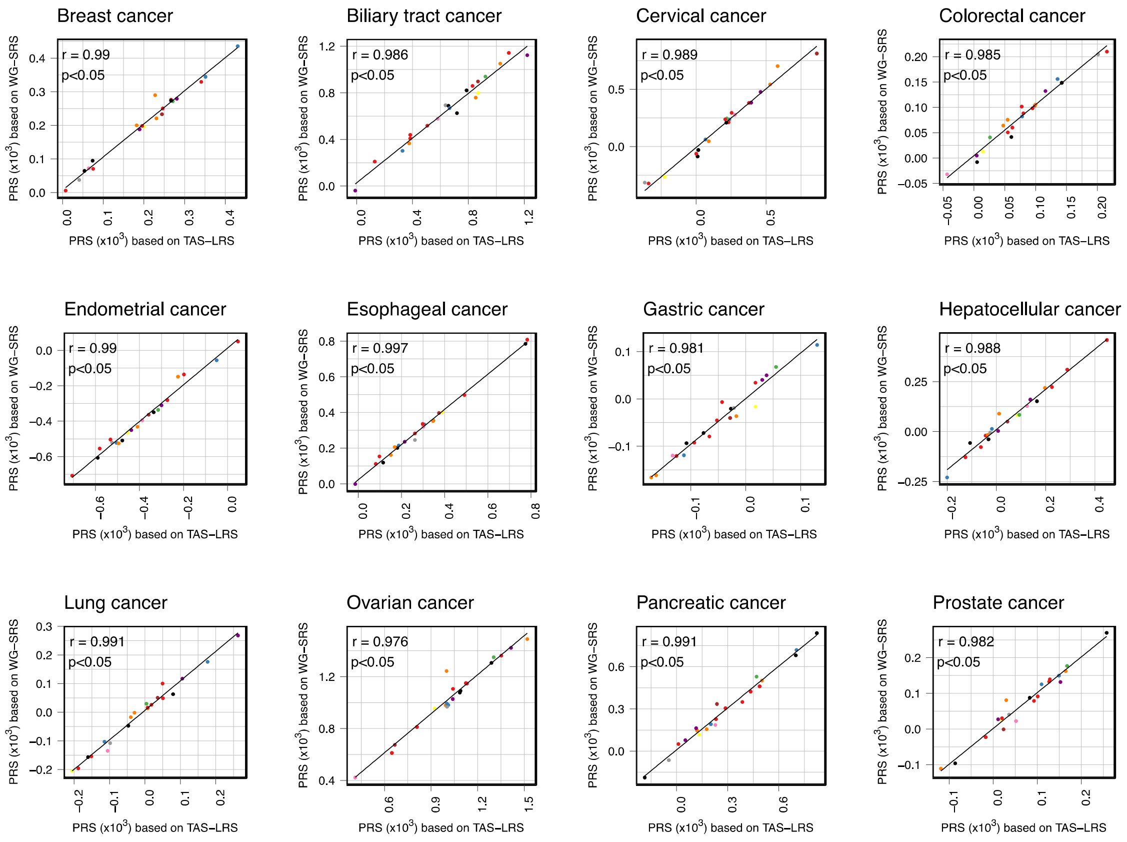

Supplementary Figure 12: **Comparison of Polygenic Risk Scores (PRSs) for 12 cancers calculated from the genotype by TAS-LRS (X-axis) and WG-SRS (Y-axis).** Each point indicates each sample and each color indicates each syndrome name (red: Familial adenomatous polyposis, blue: Familial pancreatic cancer, green: Hepatic angiomyolipoma, purple: Hereditary breast and ovarian cancer, orange: Li-Fraumeni syndrome, yellow: Lynch syndrome, brown: Multiple endocrine neoplasia type 1, pink: Multiple endocrine neoplasia type 2, gray: PTEN hamartoma tumor syndrome, black: Retinoblastoma).

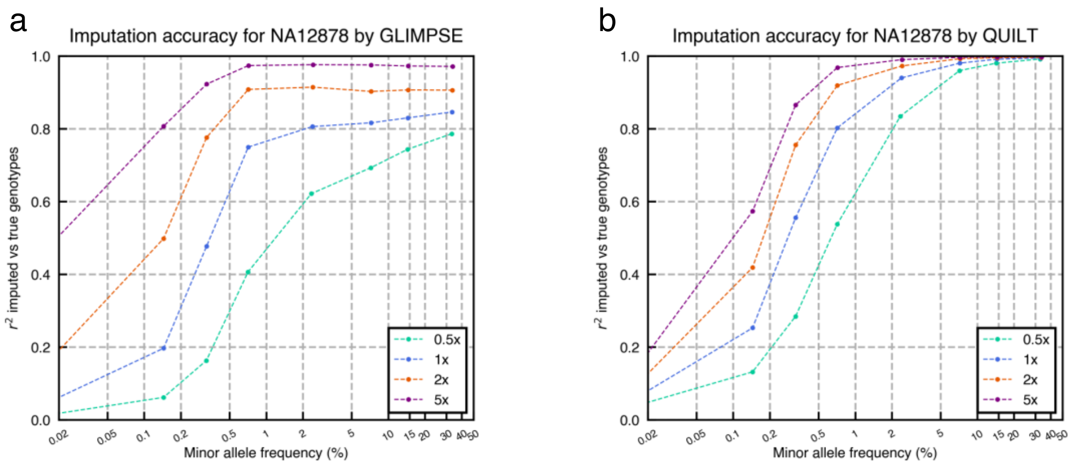

Supplementary Figure 13: Genotyping accuracy using low-coverage ONT sequencing data with phased benchmark variant (HG001 / NA12878). (a, b) Imputation accuracy of HG001 using GLIMPSE (a) and QUILT (b). Nanopore sequence data was downloaded from http://s3.amazonaws.com/nanopore-human-wgs/rel7/rel_7.fastq.gz, and downsampled. As a reference panel, we used phased variant files downloaded from the 1000 Genomes Project (s3://1000genomes/1000G_2504_high_coverage/working/20201028_3202_phased/) removing HG001 columns. The accuracy of genotype is calculated using "GLIMPSE2_concordance" against the removed HG001 genotype information. Each point represents the average of chromosomes 1 to 22 for each minor allele frequency. Each color of the line graph indicates the downsampling depth (green: 0.5x, blue: 1x, orange: 2x, purple: 5x).

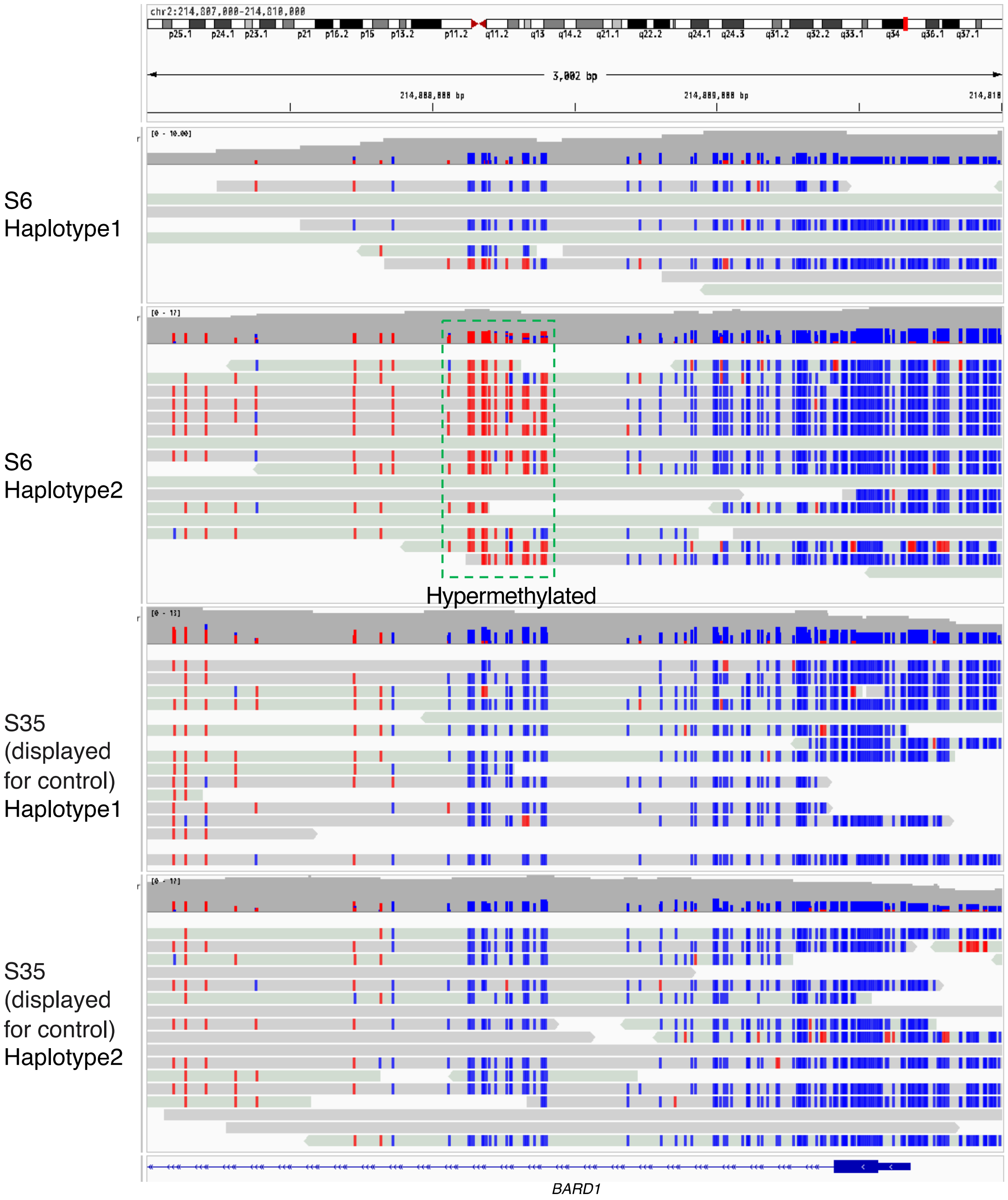

Supplementary Figure 14: A case of a possible *BARD1* epimutation. Alignment view of around the promoter region of the *BARD1* gene of the patient S6 and randomly selected control (S35). Each read was classified as haplotype 1 or 2 using Whatshap software. The CpG sites of each read are colored red if methylated and blue if not. It can be clearly seen that methylation is increased specifically for haplotype 2.
